# Biological Activities and LC–QTOF–MS-Based Phytochemical Characterization of *Onosma alboroseum* Fisch. et Mey. subsp. *alboresum* var. *alboroseum* Extracts and Extract-Loaded Nanoparticles

**DOI:** 10.3390/ph19030451

**Published:** 2026-03-11

**Authors:** Duygu Taskin, Beyzanur Ongün, Duygu Dişçi, Shalaleh Hasan Niari Niar, Fatma Betül Zengin, Erkan Rayaman, Ömer Kılıç, Turgut Taskin, Elif Çalışkan Salihi, Hatice Kübra Elçioğlu

**Affiliations:** 1 Department of Analytical Chemistry, Faculty of Pharmacy, University of Health Sciences, Istanbul 34668, Türkiye; 2 Department of Pharmacognosy, Institute of Health Sciences, Marmara University, Istanbul 34722, Türkiye; ecz.beyzanurongun@gmail.com (B.O.); fatma.zengin@marmara.edu.tr (F.B.Z.); 3 Department of Basic Pharmaceutical Sciences, Faculty of Pharmacy, Marmara University, Istanbul 34854, Türkiye; shalaleh.hniariniar@gmail.com (S.H.N.N.); elif.caliskan@marmara.edu.tr (E.Ç.S.); 4 Marmara Pharmacy Drug and Innovative Product Development Unit, Faculty of Pharmacy, Marmara University, Istanbul 34854, Türkiye; erayaman@marmara.edu.tr (E.R.); turguttaskin@marmara.edu.tr (T.T.); kubra.elcioglu@marmara.edu.tr (H.K.E.); 5 Department of Pharmacognosy, Faculty of Pharmacy, Marmara University, Istanbul 34854, Türkiye; 6 Department of Pharmaceutical Microbiology, Faculty of Pharmacy, Marmara University, Istanbul 34854, Türkiye; 7 Department of Pharmaceutical Botany, Faculty of Pharmacy, Adıyaman University, Adıyaman 02040, Türkiye; okilic@adiyaman.edu.tr; 8 Department of Pharmacology, Faculty of Pharmacy, Marmara University, Istanbul 34854, Türkiye

**Keywords:** *Onosma alboroseum*, antioxidant activity, antimicrobial activity, enzyme inhibition, LC–MS, nanoparticles

## Abstract

**Background/Objectives:** This study provides the first comprehensive evaluation of the antioxidant, antimicrobial, and enzyme inhibitory activities of *Onosma alboroseum* subsp. *alboroseum* var. *alboroseum*, including a novel nanoformulation-based comparative assessment of its most active extract. The study further aimed to investigate whether nanoparticles modulate the biological performance of the extract. **Methods:** Antioxidant activity was assessed using DPPH, FRAP, and CUPRAC assays, and total phenolic content was determined by the Folin–Ciocalteu method. Antimicrobial activity was evaluated using agar well diffusion and microdilution assays, while enzyme inhibitory activities were assessed through anticholinesterase and anti-urease assays. The most biologically active extract was subjected to LC–QTOF–MS-based tentative metabolite profiling and subsequently formulated into nanoparticles for comparative biological evaluation. **Results:** Among the extracts studied, the methanol extract had the highest total phenolic content and demonstrated superior antioxidant, antimicrobial, and enzyme inhibitor activities. LC–QTOF–MS profiling indicated a phenolic-rich composition, with rosmarinic acid as the predominant compound based on relative peak area. The methanol extract was encapsulated within alginate nanoparticles for subsequent comparative biological assessment. While the crude extract showed superior activity in antioxidant assays, nanoparticles enhanced cholinesterase and urease inhibition (28.03% and 12.11%, respectively) and improved antibacterial efficacy in microdilution assays (MIC range: 3.13–12.5 µg/mL), although no inhibition was observed in agar diffusion tests. **Conclusions:** These findings indicate the first time that the methanol extract of *Onosma alboroseum* subsp. *alboroseum* var. *alboroseum* represents a phenolic-rich source of bioactive constituents and a nanoparticle formulation that can modulate specific biological activities depending on the assay system, highlighting the relevance of formulation strategy in phytochemical-based pharmaceutical applications.

## 1. Introduction

Medicinal plants have long served as a source of therapeutic agents, largely due to their diverse primary and secondary metabolites. Among these, phenolics, alkaloids, and naphthoquinones are recognized as bioactive constituents with antioxidant, antimicrobial, and enzyme inhibitory properties [1,2,3]. For this reason, research on plants and the compounds they contain is increasing and diversifying [4].

Free radicals are atoms or molecules that contain at least one unpaired electron in their outer orbit and are therefore reactive. In biological systems, oxygen-derived (reactive oxygen species) and nitrogen-derived (reactive nitrogen species) free radicals are produced. Reactive oxygen species are formed as by-products (endogenous) during reduction and oxidation reactions occurring in the cell or due to exogenous factors such as drugs, environmental pollutants, alcohol, and radiation [5,6].

Compounds that destroy, sweep, reverse their behaviour or suppress the effects of reactive oxygen species are called antioxidants. Oxidative stress occurs when the balance between the amounts of free radicals and antioxidants is not maintained and the rates of reactive oxygen species increase in the organism. Oxidative stress can cause various negative effects, such as lipid peroxidation, deoxyribonucleic acid (DNA) and protein destruction in healthy cells in the body [7]. For this reason, oxidative stress contributes to the pathogenesis of diseases such as cancer, cardiovascular diseases, diabetes, neurological disorders (Alzheimer’s disease) and the aging process [8]. Therefore, identifying plant-derived compounds capable of modulating oxidative stress and enzyme-related pathways remains a significant research focus.

The misuse of antibiotics, which are of great importance for the treatment of infectious diseases, leads to the formation of antibiotic resistance over time. Plants, which are one of the subjects of research to find solutions to this situation, have been used as traditional folk remedies for the treatment of various infectious diseases from the past to the present. Antimicrobial activity studies with plants shed light on the discovery of potential active substances [9].

In parallel, enzymes such as acetylcholinesterase (AChE) and urease are important pharmacological targets. AChE inhibitors are relevant in the management of neurodegenerative conditions such as Alzheimer’s disease, whereas urease inhibitors may help control urease-producing pathogens associated with gastrointestinal infections. Despite the ethnomedicinal relevance of many plant taxa, enzyme inhibition profiles remain insufficiently characterized for numerous species [10,11].

However, the direct application of crude plant extracts presents several limitations. Phenolic compounds are prone to oxidative degradation, many bioactive constituents exhibit poor aqueous solubility, and overall bioavailability may be inconsistent due to rapid metabolism or instability. To address these limitations, nanoparticle-based delivery systems have gained attention [12,13].

Nanoparticles can enhance physicochemical stability, enable controlled release, improve solubility of hydrophobic constituents, and potentially modulate biological activity by altering release kinetics and interaction profiles [14,15]. In this context, plant-derived or biocompatible nanoparticle systems offer advantages such as biodegradability and reduced toxicity [16].

The genus *Onosma* belongs to the Boraginaceae family and is a genus with 230 species in the world, although it is generally distributed in Central Asia and Mediterranean countries. *Onosma* L. represents more than 105 taxa in the flora of Türkiye, and its endemism rate (52%) is quite high [10,11]. *Onosma* taxa have remarkable ethnobotanical properties, many of which are traditionally used in inflammatory diseases, pain, fever and wound treatment. Studies indicate that *Onosma* species have a rich content of alkaloids, naphthoquinones and phenolic substances [17,18].

Previous studies with *Onosma alboroseum* subsp. *alboroseum* var. *alboroseum* revealed that the plant has antioxidant activity, cytotoxic activity on various cell lines, DNA topoisomerase II inhibitory activity and tyrosinase enzyme inhibitory activity [1,19]. A literature review revealed no studies on the anticholinesterase, antiurease, and antimicrobial effects of different extracts of the *Onosma alboroseum* subsp. *alboroseum* var. *alboroseum* or on the production of bioactive extract-loaded nanoparticles.

To systematically explore phytochemical diversity, sequential extraction using petroleum ether, chloroform, and methanol was employed to generate fractions across a polarity gradient. This approach enables the selective enrichment of nonpolar constituents (e.g., lipophilic naphthoquinones), moderately polar compounds (e.g., certain alkaloids), and polar phenolic substances. Such fractionation enhances the likelihood of correlating biological activities with specific phytochemical classes.

Therefore, the crude extracts of *Onosma alboroseum* subsp. *alboroseum* var. *alboroseum* were evaluated for antioxidant, antimicrobial, and enzyme inhibitory activities. The most bioactive extract was characterized by LC–MS and formulated into nanoparticles, allowing comparison of the biological activities of the free extract and the extract-loaded nanoparticles. This approach integrates chemical profiling with pharmacological evaluation, providing a comprehensive understanding of the therapeutic potential of *Onosma alboroseum* subsp. *alboroseum* var. *alboroseum* extracts.

## 2. Results

### 2.1. Antimicrobial Activities of the Extracts and the Extract-Loaded Nanoformulations

**Figure 1:**
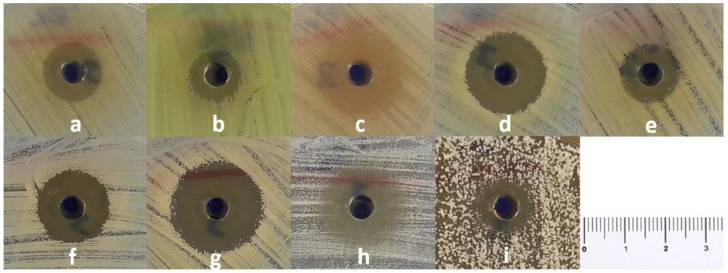
Antimicrobial activity of the methanol extract of *O. alboroseum* determined by the agar well diffusion method ((**a**): *E. coli* ATCC 25922, (**b**): *P. aeruginosa* ATCC 27853, (**c**): *P. mirabilis* ATCC 14153, (**d**): *A. baumannii* ATCC 19606, (**e**): *K. pneumoniae* ATCC 4352, (**f**): *S. aureus* ATCC 25923, (**g**): *S. aureus* ATCC 43300, (**h**): *S. epidermidis* ATCC 12228, (**i**): *C. albicans* ATCC 90028).

The methanol extract exhibited relatively high antimicrobial activity against all tested microorganisms except *Enterococcus faecalis* (Figure 1). Notably, the methanol extract exhibited lower MIC values compared to the other extracts. The petroleum ether and chloroform extracts showed activity against *Staphylococcus epidermidis*, *Acinetobacter baumannii*, and *Candida albicans*. Methanolic extract-loaded nanoparticles did not show antimicrobial activity when evaluated using the agar well diffusion method; however, antimicrobial activity was detected by the broth microdilution method against *Staphylococcus aureus* ATCC 43300, *S. epidermidis*, *Pseudomonas aeruginosa*, *Proteus mirabilis*, *A. baumannii*, *Klebsiella pneumoniae*, and *C. albicans* (Table 1). No antimicrobial activity was detected in the nanoparticles without extract using either method.

### 2.2. Antioxidant Activities and Total Phenolic Content of the Extracts and Nanoformulations

The antioxidant capacities of petroleum ether, chloroform, and methanol extracts were evaluated using DPPH, FRAP, and CUPRAC assays (Table 2). Across all assays, the methanol extract consistently exhibited the strongest antioxidant performance, whereas the petroleum ether extract showed negligible or weak activity. All extracts demonstrated lower activity compared to the respective reference standards.

A similar trend was observed when comparing the crude methanol extract with its nanoparticle formulation. In all three assays, the extract-loaded nanoparticles displayed reduced antioxidant capacity relative to the free extract. In the DPPH assay, this was reflected by a higher IC_50_ value for the nanoparticle formulation, indicating diminished radical scavenging efficiency. Likewise, FRAP and CUPRAC values of the nanoformulated extract were markedly lower than those of the crude methanol extract.

Empty nanoparticles did not exhibit detectable DPPH or CUPRAC activity; however, a minor ferric reducing capacity was observed in the FRAP assay. This weak response may be attributed to non-phenolic reducing functionalities associated with the alginate matrix or residual crosslinking ions, although its magnitude was negligible compared to the methanol extract.

Total phenolic content analysis revealed that the methanol extract contained the highest level of phenolic compounds, whereas other extracts showed considerably lower amounts. No measurable phenolic content was detected in either empty or extract-loaded nanoparticles, consistent with the absence of free phenolic compounds in the nanoparticle matrix.

Overall, the parallel trend observed between total phenolic content and antioxidant performance supports the contribution of phenolic constituents to the redox activity of the methanol extract. The findings indicate a strong correlation between total phenolic content and antioxidant test results.

### 2.3. Enzyme Inhibitory Activities (Anticholinesterase and Anti-Urease) of the Extracts and Extract-Loaded Nanoparticles

The acetylcholinesterase inhibitory activities of extracts, standards and nanoparticles were comparatively evaluated at a concentration of 100 μg/mL using the modified Ellman method. Galantamine was used as the standard substance. According to the results obtained, the methanol extract was determined to have the highest anticholinesterase activity compared to the other extracts. It was determined that the methanol extract-loaded nanoparticle had a stronger acetylcholinesterase enzyme inhibition potential than the crude methanol extract. The fact that empty nanoparticles showed a certain degree of enzyme inhibition activity suggests that it may be due to the alginate present in the nanoparticles, because alginate is a natural polymer and is known to inhibit enzymes [20].

It was determined that the extracts and nanoparticles have a lower enzyme inhibition potential than the galantamine compound used as a standard.

The urease inhibitory activities of the extracts, standards and the nanoparticle formulations were determined at a concentration of 25 µg/mL using the indophenol method. Thiourea was used as the reference standard. It was found that the methanol extract had a higher urease enzyme inhibition potential compared to the other extracts. The nanoparticle obtained by loading with the methanol extract was found to have stronger anti-urease activity than the crude methanol extract. A certain degree of enzyme inhibitory effect was observed in the empty nanoparticle, suggesting that this effect originated from the natural polymer alginate. Nanoformulation increased acetylcholinesterase inhibition by 1.74-fold (74%) compared to the free methanol extract, whereas urease inhibition showed a 1.15-fold (14.9%) increase. Furthermore, extract-loaded nanoparticles exhibited 2.72-fold higher AChE inhibition and 1.48-fold higher urease inhibition relative to empty nanoparticles. These quantitative differences suggest that the enhanced activity is mainly attributable to the loaded phytoconstituents, with the magnitude of potentiation varying between enzyme systems. The extracts and formulations were found to have lower enzyme inhibition values than the thiourea compound (Table 3).

### 2.4. LC–MS Characterization of the Methanol Extract

**Figure 2:**
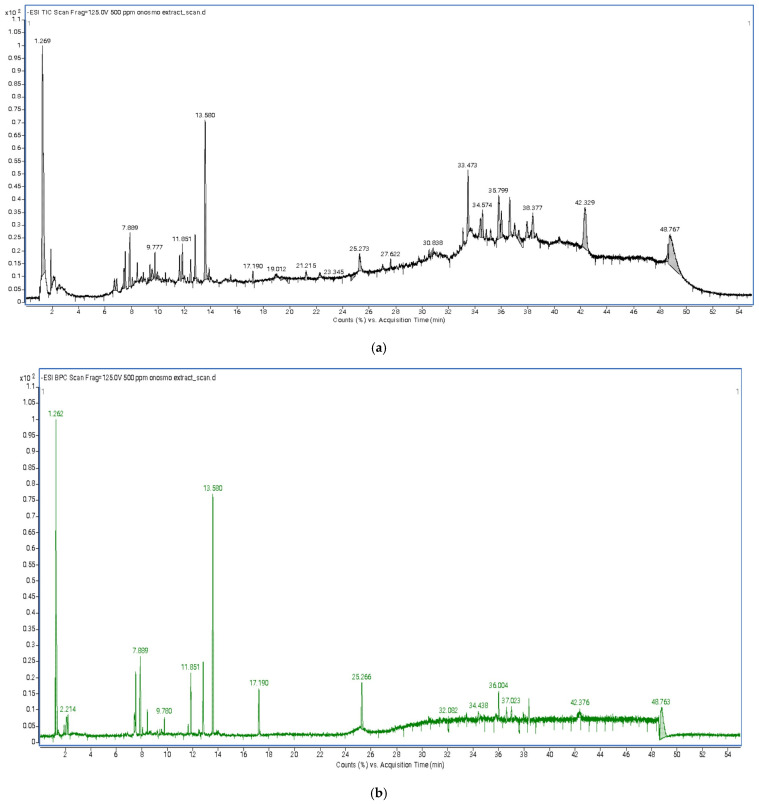
(**a**) Total ion chromatogram (TIC) obtained from LC–MS analysis of the methanol extract of *Onosma alboroseum* subsp. *alboroseum* var. *alboroseum*. (**b**) Base peak chromatogram (BPC) obtained from LC–ESI–MS analysis of the methanol extract of *Onosma alboroseum* subsp. *alboroseum* var. *alboroseum*.

The methanolic extract of *Onosma alboroseum* subsp. *alboroseum* var. *alboroseum* was analyzed using an LC–ESI–QTOF–MS system operating in negative ionization mode under full-scan acquisition. Total ion chromatograms (TICs) and base peak chromatograms (BPCs) were recorded over the *m*/*z* range 100–1100 for comprehensive metabolite profiling. Compound annotation was carried out using the Find by Formula algorithm based on high-resolution, accurate precursor ion masses (mass error ≤ 5 ppm) and isotopic pattern matching. Tentative identification was achieved through comparison with the METLIN Metabolite and Chemical Entity Database. For reporting, compounds were selected based on a database matching score ≥ 90%, mass accuracy within ±5 ppm, and isotopic pattern consistency. After applying these filtering criteria, 24 metabolites with the highest confidence scores were retained and are presented as tentative identifications. As no targeted MS/MS acquisition was performed, compound assignments are based exclusively on accurate mass and isotopic pattern information and therefore correspond to high-confidence tentative identifications. Representative full-scan mass spectra of selected compounds are provided in the Supplementary Materials (Figures S1–S24). Semi-quantitative evaluation based on normalized relative peak areas indicated that phenolic acids and flavonoid derivatives were the predominant chemical classes. Among the detected constituents, rosmarinic acid exhibited the highest relative abundance, supporting the phenolic-rich profile of the methanol extract and providing a plausible chemical basis for its observed biological activities. Comprehensive information on annotated metabolites, including retention times, proposed molecular formulas, detected ion species, mass errors, and relative peak areas, is presented in Figure 2a,b and Table 4.

### 2.5. Production and Physicochemical Characterization of the Extract-Loaded Nanoparticles

**Figure 3:**
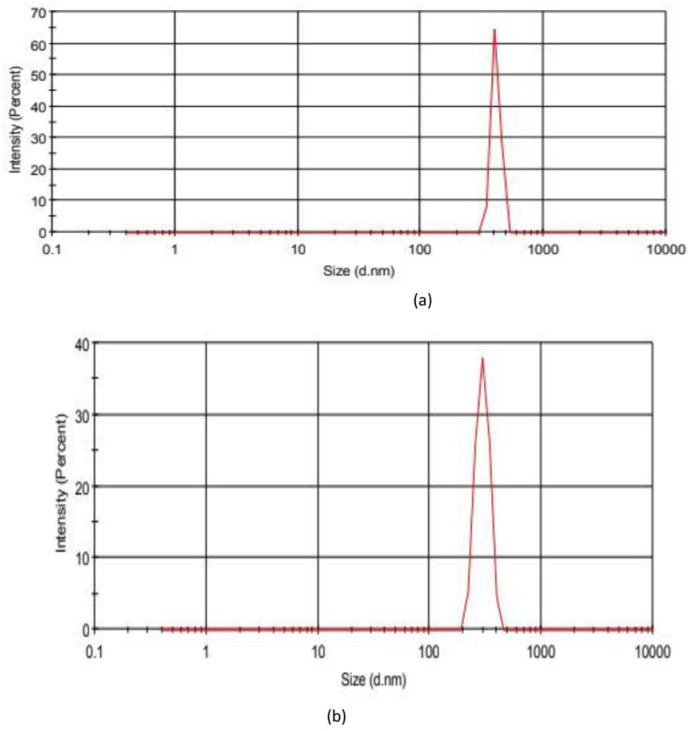
Particle size distribution plots of methanol extract-loaded nanoparticles (**a**) and empty nanoparticles (**b**).

Nanoparticle formulations were produced by using the methanol extract of the *Onosma alboroseum* subsp. *alboroseum* var. *alboroseum,* which has the highest bioactivity among the extracts prepared. Nanoparticles were produced in accordance with green chemistry principles by using alginate, a natural polymer, as a matrix without using any additives, surface active agents or solvents other than distilled water and drying the resulting gel beads under ambient pressure [21]. For the characterization of the nanoparticles produced, firstly, particle size distributions were examined by Zetasizer using the dynamic light scattering (DLS) method. The particle size distributions of the prepared dispersions were measured and the mean hydrodynamic particle sizes were found to be 409.0 nm (SD 33.93) and 297.7 nm (SD 41.88) for the extract-loaded nanoparticles and empty nanoparticles, respectively. The results obtained (Figure 3) confirmed that the formulations produced were nanosized particles with heterogeneity according to the reasonable PDI values, which are 0.669 and 0.633 for the extract-loaded nanoparticles and empty nanoparticles, respectively [22]. Secondly, encapsulation efficiency (EE, %) and extract loading capacity (LC, %) were calculated for the extract-loaded nanoparticles by the spectrophotometric method using previously prepared calibration curves and equations given in Section 4 [22]. The results obtained are shown in Table 5. As seen from the table, extract-loaded nanoparticles have a modest encapsulation efficiency in correlation with the previous studies in the literature. Alginate is the most commonly used biopolymer in ionic gelation methods; however, the high porosity of the network structure of the gel produced with alginate shows moderate loading efficiencies [23].

**Figure 4:**
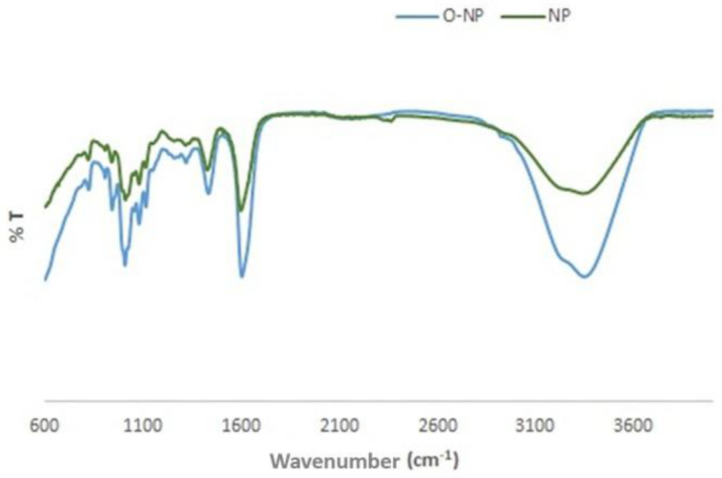
FTIR spectra (4000–600 cm^−1^) of extract-loaded nanoparticles (O-NPs) and empty nanoparticles (NPs).

FTIR spectra were taken in the range of 600–4000 cm^−1^ using an FTIR (Fourier Transform Infrared) spectrophotometer in order to determine the chemical bonds and functional groups in the structure of the nanoparticles produced (Figure 4). In the FTIR spectra in Figure 4, hydroxyl groups of the alginate structure are seen at 3300 cm^−1^, asymmetric and symmetric vibrations of COO^−^ groups are seen at 1600 cm^−1^ and 1400 cm^−1^ and C-O-C bonds are seen at 1000 cm^−1^ [24,25]. The FTIR spectra showed that the nanoparticles were successfully produced, and the extract encapsulated by the alginate structure was physically dispersed in the nanoparticle matrix.

**Figure 5:**
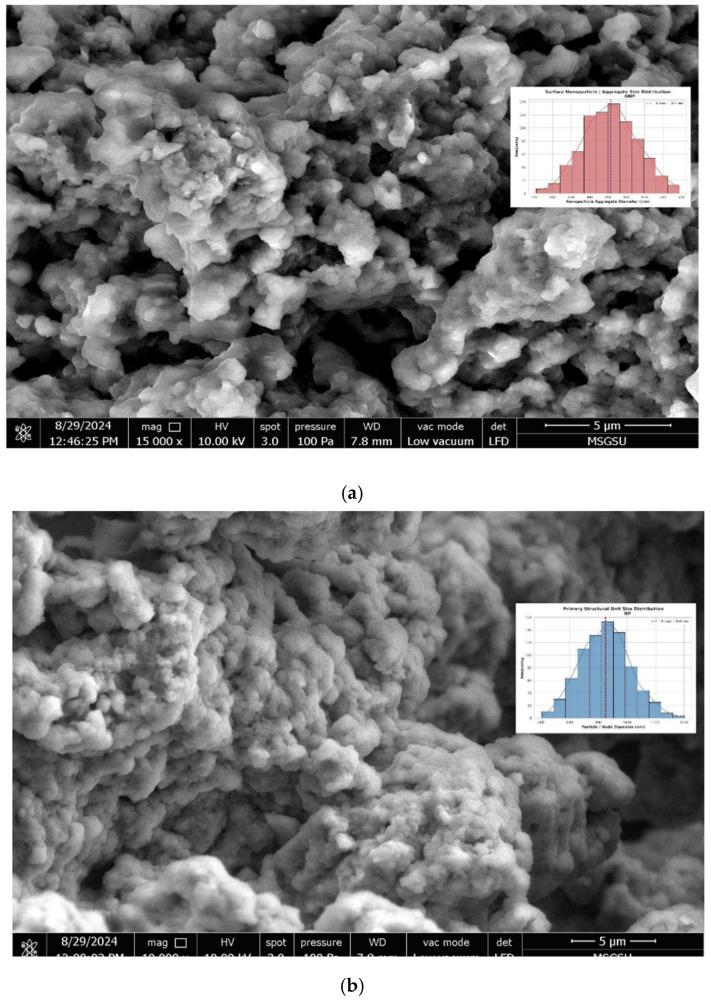
SEM micrographs of extract-loaded nanoparticles (**a**) and empty nanoparticles (**b**).

Scanning electron microscopy was used to visualize the shape and the surface properties of the produced nanoparticles. Image analysis was also done and given in the figures for the interpretation of the nanoparticle sizes. As can be seen from the SEM photographs (Figure 5), the produced formulations are nano-sized and have heterogeneous spherical shapes [26].

**Figure 6:**
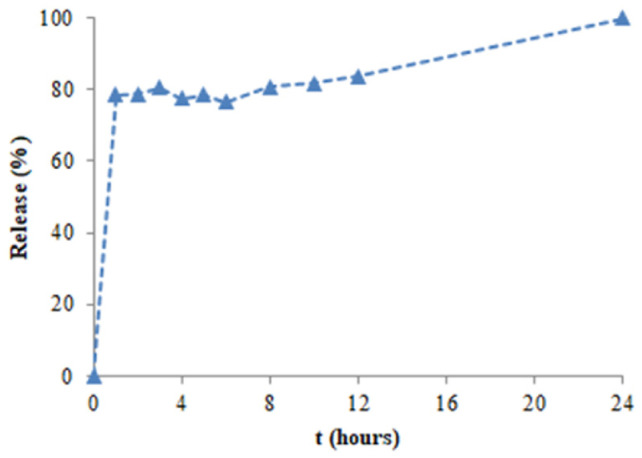
In vitro release profile of the extract-loaded nanoparticles.

In vitro release abilities of the *Onosma alboroseum* subsp. *alboroseum* var. *alboroseum*-encapsulated nanoparticles were studied in PBS medium (pH 7.4) at 37 °C to show their capability to be used in drug delivery applications. Release profiles of the extract-loaded nanoparticles are shown in Figure 6. There is a burst release effect in the first hour due to alginate’s relatively loose and mesh structure and the physical nature of its drug retention mechanism, which is followed by a gradual release in the following hours [27]. In total, 80% of the extract was released from the nanoparticle matrix in the first 3 h, while the release of the extract continued gradually from the nanoparticle matrix in the next hours, and 100% of the extract was released in 24 h [28]. The release profile of the extract-loaded nanoparticles showed that the formulations produced are promising natural drug delivery systems synthesized using the green chemistry approach [28].

## 3. Discussion

Previous studies on *Onosma alboroseum* subsp. *alboroseum* var. *alboroseum* revealed that the plant has antioxidant activity, cytotoxic activity on various cell lines, DNA topoisomerase II inhibitory activity and tyrosinase enzyme inhibitory activity [1,20]. In this study, the antioxidant and antimicrobial activities of petroleum ether, chloroform and methanol extracts of *Onosma alboroseum* subsp. *alboroseum* var. *alboroseum* was investigated. Alginate nanoparticles loaded with extracts showing stronger biological activity were prepared.

The bioactivity results of the prepared nanoparticle formulation were compared with those of the crude extract. It was determined that the methanol extract obtained from *Onosma alboroseum* subsp. *alboroseum* var. *alboroseum* showed stronger antioxidant activity (DPPH (IC_50_: 0.036 mg/mL), FRAP (2.256 mM FeSO_4_/mg extract) and CUPRAC (2.595 mM Trolox equivalent/mg extract)) than petroleum ether and chloroform extracts. According to the FCR results, it was determined that the methanol extract contained the highest amount of phenolic substances (0.080 mg GAE/mg extract) compared to the other extracts.

In enzyme inhibition assays, the methanol extract exhibited moderate acetylcholinesterase (16.1%) and urease (10.5%) inhibitory activities at the tested concentrations, outperforming petroleum ether and chloroform extracts. Notably, methanol extract-loaded nanoparticles demonstrated substantially enhanced inhibition (28.0% and 12.1%, respectively), suggesting that nanoparticles may facilitate improved interaction of bioactive compounds with enzyme active sites. Nanoparticles selectively enhanced the enzyme inhibitory activity of the methanol extract. For acetylcholinesterase, the nanoformulation resulted in a 1.74-fold (74%) increase in inhibition compared to the free extract, demonstrating a significant improvement in biological activity. Furthermore, extract-loaded nanoparticles exhibited 2.72-fold higher acetylcholinesterase inhibition compared to empty nanoparticles, indicating that the observed activity was primarily due to the encapsulated phytochemicals rather than the carrier matrix. This improvement may be explained by the larger surface area of the nanoparticles, better dispersion of the extract, enhanced stability of the bioactive compounds, and more effective interaction with the enzyme’s active site, as reported in previous nanoformulation-based anticholinesterase studies [29].

In contrast, urease inhibition showed a relatively modest improvement following nanoparticles. The nanoformulation resulted in a 1.15-fold (14.9%) increase compared to the free extract and a 1.48-fold increase compared to empty nanoparticles. However, given the relatively low absolute inhibition levels and the measurable activity of the carrier system itself, the contribution of encapsulated phytochemicals to urease inhibition appears more limited. This difference in activity may be due to structural differences between acetylcholinesterase and urease, particularly in the shape and accessibility of their active sites and in how ligands interact with these sites [30,31].

Overall, these findings suggest that nanoparticles selectively potentiate enzyme inhibitory activity, particularly against acetylcholinesterase, and may represent a promising strategy to improve the bioefficacy of plant-derived inhibitors.

In the determination of antimicrobial activity within the scope of this study, samples of 60 mg/mL concentration of petroleum ether, chloroform and methanol extracts of the plant were prepared and tested using the agar well diffusion method and microdilution methods. In our study, particular emphasis was placed on the selection of clinically significant microorganisms capable of causing infections in humans. For this purpose, nine bacterial strains and one yeast strain were employed in the present study. Petroleum ether and chloroform extracts of the plant showed antimicrobial activity against *S. epidermidis*, *A. baumanii*, and *C. albicans*. The methanol extract exhibited antimicrobial activity against all microorganisms except *E. faecalis* and was identified as the most potent extract in terms of antimicrobial efficacy among all the tested extracts (Table 1).

Investigations into the antimicrobial activity of *Onosma* species are limited. It has been reported that the ethanol extracts of *Onosma alboroseum* Fisch. & Mey. subsp. *albo-roseum* var. *alboroseum*, *Onosma molle* DC., *Onosma sericeum* Willd., and *Onosma auriculatum* Aucher ex DC. against *Bacillus cereus*, *Bacillus subtilis*, *Enterobacter aerogenes*, *E. coli*, *K. pneumoniae*, *P. aeruginosa*, *S. aureus*, *Candida* sp., and *Saccharomyces cerevisiae* [32]; the ethanol, methanol, and ethyl acetate extracts of the aerial parts and roots of *Onosma* nana against *B. cereus*, *E. coli*, *Proteus vulgaris*, *Listeria monocytogenes*, *S. aureus*, *S. epidermidis*, *P. aeruginosa*, and *Candida tropicalis* [33]; the methanol extract of the aerial parts of *Onosma halophila* against *Streptococcus pneumoniae*, *E. faecalis*, *E. coli*, *P. aeruginosa*, *Candida metapsilosis*, and *Candida parapsilosis* [34]; the n-hexane–dichloromethane (1:1) extract of the roots of *Onosma argentatum* against *B. subtilis*, *E. coli*, *and S. aureus* [35]; the acetone, chloroform, methanol, ethanol, and n-hexane–dichloromethane extracts of the roots of *Onosma dichroanthum* against *S. aureus*, *B. cereus*, *and Micrococcus luteus* [36]; the n-hexane, ethyl acetate, methanol, and aqueous extracts of the leaves of *Onosma bracteatum* against *S. aureus*, *E. coli*, *P. aeruginosa*, and *C. albicans* [37]; the ethanol and supercritical CO_2_ extracts of the aerial parts of *Onosma angustissima* against *S. aureus*, *E. faecalis*, *P. aeruginosa*, and *E. coli* [38] were found to exhibit antimicrobial activity. When the results of these studies are examined, it is observed that *Onosma* species exhibit antimicrobial activity [32]. In the study related to the antimicrobial activity of the species included in our research [32], the ethanol extract of *O. alboroseum* was used, and the disk diffusion method was applied to determine antimicrobial activity. The researchers did not detect any antimicrobial activity against Bacillus cereus, *B. subtilis*, *E. aerogenes*, *E. coli*, *K. pneumoniae*, *P. aeruginosa*, *S. aureus*, *Candida* sp., and *S. cerevisiae*. The absence of antimicrobial activity may be attributed to the low amount of sample applied in the disk diffusion method. In addition, differences between the extract and the plant material may have contributed to the lack of detectable antimicrobial activity, which could explain the discrepancy between their findings and the results obtained in the present study.

When the activities of the extracts against Gram-negative and Gram-positive bacteria were generally evaluated based on their MIC values, the methanol and chloroform extracts exhibited similar efficacy against both Gram-positive and Gram-negative bacteria. The methanol extract, however, was effective against a greater number of microorganisms compared to the other extracts. The MIC values of the methanol extract ranged from 3.75 to 7.5 mg/mL against Gram-negative microorganisms and from 0.94 to 3.75 mg/mL against Gram-positive microorganisms. Lower MIC values were determined for Gram-negative microorganisms (Table 1).

Due to the strong antimicrobial activity of the methanol extract, nanoparticles were synthesized from this extract, and the retention of antimicrobial activity was assessed using the same methods. While the antimicrobial activity of the nanoparticles prepared from the methanol extract could not be detected using the agar well diffusion method, they exhibited antimicrobial activity against *S. aureus*, *S. epidermidis*, *P. aeruginosa*, *P. mirabilis*, *A. baumannii*, *K. pneumoniae*, and *C. albicans* when tested using the microdilution method (Table 1). We hypothesize that the lack of antimicrobial activity observed in the agar well diffusion assay may be attributed to differences in the test medium. The solid medium used in the agar well diffusion test might have impeded the diffusion of the nanoparticles. The encapsulation efficiency was 29.7% (Table 5). Accordingly, the extract concentration in the nanoparticles tested in the antimicrobial activity assays was calculated as 6.89 mg per 100 mg of nanoparticles. Since the amount of extract present in the tested sample was lower than that of the pure extract, no inhibition zone was observed in the agar well diffusion assay. Furthermore, when the MIC values of the extract and ME-NP are examined, the MIC values reported for ME-NP in Table 1 are based on the total nanoparticle weight. The corresponding extract amounts calculated from these MIC values are 0.43 mg/mL for *P. aeruginosa*, 0.86 mg/mL for *P. mirabilis*, 0.22 mg/mL for *A. baumannii*, 0.43 mg/mL for *K. pneumoniae*, 0.22 mg/mL for *S. aureus* ATCC 43300, 0.06 mg/mL for *S. epidermidis*, and 0.12 mg/mL for *C. albicans*. No MIC value was obtained for the blank nanoparticles. Considering these values, it was determined that, except for *E. coli* and *S. aureus* ATCC 25923, the other microorganisms exhibited antimicrobial activity at lower effective extract concentrations following nanoparticle formulation (Table 1).

Phytochemical investigations conducted on various species of the *Onosma* genus consistently report a predominance of phenolic acids and flavonoids. Studies on *O. sericea*, *O. stenoloba*, and *O. angustissima* revealed diverse hydroxycinnamic acids, flavonoid glycosides, and related phenolic metabolites as major constituents [39,40]. Similarly, ESI–MS/MS profiling of endemic Turkish species such as *O. lycaonica* and *O. papillosa* demonstrated that rosmarinic acid, luteolin derivatives, apigenin glycosides, and caffeic acid derivatives are characteristic metabolites of the genus [41]. These reports collectively establish *Onosma* species as phenolic-rich taxa [42].

In agreement with the established phytochemical pattern of the genus, high-resolution LC–QTOF–MS profiling of *Onosma alboroseum* subsp. *alboroseum* var. *alboroseum* revealed a metabolite composition dominated by phenolic acids, hydroxycinnamic acid derivatives, and flavonoid glycosides. Although compound assignments are based on accurate mass and isotopic pattern data without MS/MS confirmation, the detected metabolite classes are fully consistent with previously reported profiles for related species.

Semi-quantitative analysis showed that rosmarinic acid was the most abundant compound (48.89%), which clearly showed a substantial accumulation in the plant material compared to the previously reported *Onosma* species. These differences in abundance could be due to interspecies variations in metabolism, as well as environmental factors such as altitude, soil type, and climatic conditions. It is well known that the metabolic production of phenolic compounds, especially hydroxycinnamic acid derivatives like rosmarinic acid, is significantly affected by environmental stress factors [43]. Thus, the substantial abundance in the current study could be attributed to local adaptations.

The potent antioxidant activity of the compounds shown by DPPH, FRAP, and CUPRAC assays may be mechanistically explained by the structural features of the identified phenolics. For instance, the structure of rosmarinic acid with two catechol units and four hydroxyl groups shows potent hydrogen atom transfer (HAT) and single electron transfer (SET) ability [44]. The ortho-dihydroxy group shows resonance stabilization of the resulting phenoxyl radical. Flavonoids with a catechol B-ring and a C2=C3 double bond with a 4-oxo group show enhanced antioxidant activity by electron delocalization. Although the glycosylated compounds may show slightly reduced antioxidant activity of the parent aglycones, they make a substantial contribution to the overall redox activity of the crude extract [44].

Notably, while the crude methanol extract showed superior activity in the antioxidant assays, the nanoparticle formulation showed enhanced enzyme inhibitory activity and antibacterial activity. This is possibly due to differences in bioaccessibility. In chemical assays with a short duration, the activity is mainly due to the immediate availability of freely soluble phenolic compounds. However, nanoparticles may hinder the interaction with free radicals. On the other hand, in enzyme inhibitory activity and microdilution assays for antibacterial activity, the activity is possibly enhanced due to the prolonged duration of exposure. This allows for better interaction with biological membranes or enzyme sites. This study suggests that nanoparticles are not just a simple preservation strategy but may have a modulating effect on bioactivity.

Overall, high-resolution LC–QTOF–MS profiling confirmed the phenolic-dominant composition of the methanol extract, with rosmarinic acid representing the most abundant constituent, thereby providing a coherent chemical rationale for the observed bioactivities. The predominance of this compound, together with complementary phenolic derivatives, supports the strategic selection of the extract for nanoformulation. Collectively, the findings demonstrate that nanoparticles can modulate specific biological responses of phenolic-rich plant extracts—particularly in antimicrobial and enzyme inhibition models—highlighting their potential utility in phytochemical-based pharmaceutical development.

## 4. Materials and Methods

### 4.1. Reagents, Solvents, and Materials

All chemicals and reagents used in this study were of analytical grade unless otherwise specified. Solvents including methanol, chloroform, and petroleum ether were purchased as analytical grade from local suppliers and used without further purification. Gallic acid, used as the standard for total phenolic content determination, and 2,2-diphenyl-1-picrylhydrazyl (DPPH) were obtained from Sigma-Aldrich (St. Louis, MO, USA). Ferric chloride (FeCl_3_·6H_2_O), 2,4,6-tripyridyl-s-triazine (TPTZ), and other reagents for antioxidant assays were of analytical grade. LC–QTOF–MS analyses were performed using HPLC-grade solvents purchased from Supelco (Bellefonte, PA, USA). Enzyme inhibition assays employed acetylcholinesterase and urease enzymes obtained from Sigma-Aldrich. Alginate for nanoparticle preparation was of analytical grade (Sigma-Aldrich, St. Louis, MO, USA), and calcium chloride (CaCl_2_) used as a crosslinker was also of analytical grade.

### 4.2. Plant Material and Preparation of Extracts

*Onosma alboroseum* subsp. *alboroseum* var. *alboroseum* was collected from Adıyaman (Türkiye), and was collected from the steppe-stony-rocky areas within the canyon, stretching from Tilkihan stream towards Gömükhan dam, in June 2023. The plant sample was identified by plant taxonomist Dr. Ömer KILIÇ with volume 6 book of Flora of Turkey. A herbarium sample with the plant code ÖK 4805 was deposited in Adıyaman University Faculty of Pharmacy, Department of Pharmaceutical Botany. The aerial parts of *Onosma alboroseum* subsp. *alboroseum* var. *alboroseum* were dried under room conditions. The dried plant was powdered and macerated with petroleum ether, chloroform and methanol solvents, respectively. After maceration, the liquid part was filtered through filter paper and the crude extracts were obtained by volatilization in a rotary evaporator at a temperature not exceeding 50 °C. The extracts were stored at 4 °C until the day the study began. The yields of the obtained plant extracts were determined as 2.89% in methanol extract, 0.65% in chloroform extract, and 0.67% in petroleum ether extract.

### 4.3. Tentative Characterization of Methanol Extract Constituents Using UPLC–QTOF–ESI–MS

The methanol extract was initially dissolved in LC–MS grade methanol to prepare a 1000 ppm stock solution. This solution was subsequently diluted to 500 ppm and filtered through a syringe membrane filter prior to LC–MS analysis.

An Agilent 1260 Infinity II HPLC system (Agilent Technologies, Santa Clara, CA, USA) coupled to an Agilent 6530 Accurate-Mass LC-QTOF system with an ion source ESI Agilent Jet Stream Technology was used to analyze the extracts. An Agilent 1260 Autosampler (model G7129A), a binary pump (model G7112B), and a Poroshell 120, EC-C18, 2.1 × 150 mm, 2.7 µm column were all part of the HPLC system. Separation was achieved using a binary gradient of (A) water containing 0.1% (*v*/*v*) formic acid and (B) acetonitrile at a flow rate of 0.4 mL/min and a column temperature of 35 °C. The gradient program was as follows: 2% B (0 min), 15% B (5 min), 25% B (10 min), 35% B (15 min), 40% B (20 min), 65% B (25 min), 90% B (30–45 min), returned to 2% B at 45.10 min, with a total run time of 51 min. The injection volume was 5 µL.

Mass spectrometric detection was performed in negative electrospray ionization (ESI−) mode under the following conditions: gas temperature 300 °C, drying gas flow 10 L/min, nebulizer pressure 45 psi, sheath gas temperature 350 °C, sheath gas flow 11 L/min, capillary voltage 3500 V, and fragmentor voltage 125 V. Data were acquired in full-scan MS mode over the *m*/*z* range 100–1100 at an acquisition rate of 5 spectra/s in centroid format. The mass spectrometer was externally calibrated prior to analysis to ensure mass accuracy. A mass error threshold of <5 ppm was applied during data processing.

Data acquisition was performed using MassHunter Data Acquisition software (version B.12.00), and processing was carried out using MassHunter Qualitative Analysis Workflows (version B.12.00) and Qualitative Navigator (version B.12.00). In negative ion mode, deprotonated molecules [M−H]^−^ were primarily observed. In addition, formate adduct ions ([M+HCOO]^−^) were detected for certain compounds due to the presence of formic acid in the aqueous mobile phase. Representative full-scan mass spectra of selected annotated compounds are provided in the Supplementary Materials (Figures S1–S24).

Relative peak areas were calculated by manual integration of extracted ion chromatograms (EICs) generated using theoretical *m*/*z* values with a ±5 ppm mass tolerance. The integrated peak areas of the identified compounds were summed and normalized to 100% to obtain semi-quantitative relative abundances.

### 4.4. Determination of Antimicrobial Activity

The antimicrobial activity of plant extracts and nanoparticles was determined against bacterial strains *E. coli* ATCC 25922, *S. aureus* ATCC 25923, *S. aureus* ATCC 43300, *S. epidermidis* ATCC 12228, *P. aeruginosa* ATCC 27853, *E. faecalis* ATCC 29212, *P. mirabilis* ATCC 14153, *A. baumannii* ATCC 19606, and *K. pneumoniae* ATCC 4352, and the yeast strain *C. albicans* ATCC 90028 using the agar well diffusion and microdilution methods. In the determination of antimicrobial activity, the 60 mg/mL concentration of *Onosma alboroseum* subsp. *alboroseum* var. *alboroseum* extracts in DMSO and the nanoparticles of the methanol extract were used.

#### 4.4.1. Agar Well Diffusion Method

The bacteria were cultured on tryptic soy agar (TSA), and *C. albicans* was cultured on Sabouraud dextrose agar (SDA). Microorganism suspensions were prepared from colonies on culture in 0.85% physiological saline solution (PSS). Bacterial suspensions were adjusted to a turbidity equivalent to McFarland 0.5 standard, while the yeast suspension was adjusted to McFarland 1 standard turbidity. These suspensions were taken with a sterile swab and spread on Mueller Hinton agar (MHA) for bacteria and SDA for yeast. Wells with a diameter of 5 mm were made on the medium using a sterile punch at certain intervals and 50 μL of the extracts dissolved in their solvents were placed in the wells. Meropenem (10 μg/well) for bacteria and amphotericin B (100 μg/well) for yeast, DMSO and PSS were used as controls. Petri dishes were incubated at 37 °C for 18–24 h for bacterial growth and at 35 °C for 24–48 h for yeast growth. Following incubation, the diameters of the inhibition zones were measured in millimeters using a digital caliper. All experiments were performed in triplicate, and the average values were calculated [45,46,47].

#### 4.4.2. Minimal Inhibitory Concentration (MIC) Determination for Bacteria

The minimal inhibitory concentration (MIC) of extracts and nanoparticles was determined using the microdilution method in accordance with the methods recommended by CLSI (formerly NCCLS). Briefly, Mueller Hinton broth was used for bacteria, and RPMI 1640 medium was used for the yeast. The media were distributed into microplates, and serial dilutions were prepared by adding the extracts and NPs. The bacteria were cultured on TSA, and *C. albicans* was cultured on SDA. Bacterial suspensions were prepared in PBS at a concentration of 5 × 10^5^ CFU/mL, and yeast suspensions were prepared in RPMI 1640 at 5 × 10^3^ CFU/mL. Subsequently, 5 µL of the bacterial suspension and 100 µL of the yeast suspension were added to the respective wells. The microplates were incubated at 37 °C for 24 h for bacteria, and at 35 °C for 24–48 h for yeast. At the end of incubation, the lowest sample concentrations without growth were determined as MIC. MHB, RPMI 1640, meropenem, amphotericin B and DMSO were used as controls [48,49]. In the determination of minimum bactericidal and fungicidal concentration (MBC, MFC, respectively), 10 µL was taken from the wells where no growth was observed, and inoculation to tryptic soy agar medium and incubated at 37 °C for 24 h. After incubation, the lowest concentration at which no growth was observed was determined as the MBC and MFC.

### 4.5. Evaluation of Antioxidant Activity

#### 4.5.1. DPPH Assay

The free radical capture efficiency of the extracts was determined using DPPH radical [50]. In total, 10 μL of the extracts prepared at concentrations of 0.5–5 mg/mL were taken and 240 μL of 0.1 mM DPPH solution was added. Extracts and standard solutions to which DPPH solution was added were kept at room temperature in the dark for 30 min. Their absorbance was measured against the reference using a microplate reader at 517 nm. The sample and control were prepared under the same conditions using 10 μL of methanol instead of the standard substance. The absorbance of the control was measured daily. The experiment was repeated three times and averaged. Before calculating the IC_50_ value, the % DPPH radical scavenging capacity was calculated with the formula given below:

% DPPH radical scavenging capacity = [(A_0_ − A_1_)/A_0_] × 100]

where A_0_ is the absorbance of the control solution and A_1_ is the absorbance of plant extracts and standard solutions.

The extract and standard substance concentration resulting in a 50% reduction in baseline DPPH• concentration is defined as IC_50_. This value was calculated using the correct equation obtained by placing % free radical removal activity values against the studied concentrations, and the results were given as IC_50_ = mg/mL.

#### 4.5.2. FRAP Assay

FRAP reagent was prepared by mixing 25 mL of 300 mM acetate buffer (pH 3.6), 2.5 mL of TPTZ solution (solution of 10 mM TPTZ at 40 mM HCl) and 2.5 mL of 20 mM FeCl_3_·6H_2_O and kept at 37 °C for 30 min. By mixing 190 μL of FRAP reagent with 10 μL of extract, replacing the extract with acetate buffer, the absorbance increase against the prepared reference was measured at 593 nm at the fourth minute. The absorbance values of the extracts at 593 nm were compared with the values of the calibration chart prepared with FeSO_4_.7H_2_O and the FRAP value was expressed as mM FeSO_4_/mg extract [51].

#### 4.5.3. CUPRAC Assay

In total, 60 μL of extracts was taken into microplate wells. In total, 60 μL of 10 mM copper (II) solution, 60 μL of 7.5 mM neocuproine solution, and 60 μL of 1.0 mM ammonium acetate (NH_4_Ac) solution were added to it, respectively. Then, 10 μL of distilled water was added. After the mixture was kept at room temperature for 60 min, its absorbance was measured at 450 nm using a reference versus microplate reader. CUPRAC values of the extracts were given as mM trolox/mg extracts [29].

#### 4.5.4. Total Phenolic Content

In total, 25 μL of extract prepared in various concentrations was taken into the microplate wells. The Folin–Ciocalteu reagent was diluted with distilled water in a ratio of 1:3 and 100 μL was added to the wells. Four minutes later, 75 μL of 2% sodium carbonate solution was added. After 2 h of keeping in room conditions, the resulting blue absorbance was measured at 765 nm against the reference. Total phenolic substance determination was applied to gallic acid solutions prepared at various concentrations with the Folin–Ciocalteu reagent. The calibration curve was prepared by graphing the concentrations against the measured absorbances, and the correct equation was obtained. From the line equation obtained, the total amount of phenolic substances of the samples was calculated as mg gallic acid (mgGAE/mg extract) equivalent. The experiment was repeated three times and averaged [29].

### 4.6. Determination of Anticholinesterase Activity

For the determination of acetylcholinesterase (AChE) inhibition activity, acetylcholinesterase enzyme obtained from electric fish was used as the enzyme, and acetyl thiocholine iodide was used as the substrate. For the measurement of activity, yellow colored 5,5- dithiobis-(2-nitrobenzoic acid) (DTNB) was used. Buffer (Tris HCl buffer) was used as the control and galantamine, an alkaloid-type drug isolated from the Galanthus plant, was used as the standard [29]. Acetylcholinesterase inhibition activity was measured using a microplate reader. In total, 20 μL of the extracts and 20 μL of the AChE enzyme solution were added to 40 μL of 50 mM Tris HCl buffer solution (pH = 8). This solution was incubated at 25 °C for 10 min. After incubation, 20 μL of substrate (acetyl thiocholine iodide) was added and incubated at 25 °C for 5 min. Then, 100 μL of DTNB reagent was added. The yellow colored 5-thio-2-nitrobenzoic acid anion formed by the reaction of thiocholine released by the hydrolysis of the substrate with DTNB was read at a wavelength of 412 nm. Acetylcholinesterase inhibitory activity was calculated using the following equation:

% Acetylcholinesterase Inhibition = [(Acontrol − Asample)/Acontrol] × 100

where Acontrol represents the absorbance of the control reaction and Asample represents the absorbance in the presence of the test sample.

### 4.7. Determination of Anti-Urease Activity

In total, 25 μL of urease enzyme was added to the microplate wells, and 10 μL of the plant extract was added and kept at 37 ^ο^C for 15 min. Then, 50 μL of urea was added and kept at 37 ^ο^C for 15 min. At the end of this time, R1 (23 μL 5% phenol + 23 μL 5 mM sodium nitroprusside) and R2 (35 μL 400 mM sodium hydroxide, 35 μL 10 mM sodium hypochlorite) were added, respectively, and kept in the incubator at 37 ^ο^C for 50 min. Buffer (EDTA, pH 8.20) was used instead of extract as a control, urease enzyme and buffer were used instead of extract as a blind, and other procedures were followed exactly. Absorbance was measured at 625 nm against a reference using a microplate reader. The percentage inhibition was calculated according to the following formula [52].

% Urease inhibition = [(Acontrol − Asample)/Acontrol] × 100

where Acontrol represents the absorbance of the control reaction and Asample represents the absorbance measured in the presence of the test sample.

### 4.8. Preparation of Extract-Loaded Nanoparticles

Nanoparticle formulations of the extract, which were found to show the highest biological activity, were prepared. In this study, extract-loaded and empty nanoparticles with aerogel structure were produced using the ionic gelation method modified in our laboratory [25,53]. In the production of nanoparticles, alginate, a natural polymer, was used in accordance with the principles of green chemistry, and the production was carried out without the addition of any additives or surfactants. For the drying of nanoparticles produced in aqueous media, methods that significantly increase energy consumption were eliminated from the process, the drying method at ambient pressure was selected, and nanosized particles were obtained in an aerogel structure with slow ambient pressure drying [54].

First, a solution of 100 mg plant extract in 10 mL of distilled water was prepared using an ultrasonic bath. In total, 01 g of sodium alginate was added to 50 mL of distilled water in a beaker and stirred with the magnetic stirrer. After complete dissolution, 10 mL of the solution was taken into a separate beaker, and the solution containing the plant extract was slowly added and stirred for 15 min. This mixture was added dropwise to a solution of calcium chloride (3 g/100 mL), and the formation of bead-shaped gels was observed. The mixture containing the gels was left to mature overnight in the dark. The next day, the gels were filtered, washed with distilled water and allowed to dry at room temperature and ambient pressure for 48 h. After this slow drying process, they were dried in an oven at 70 °C for 4 h. The obtained nanoparticles were stored in sealed containers. Empty nanoparticles were also prepared by the same method without the use of plant extract.

### 4.9. Physicochemical Characterization of Extract-Loaded Nanoparticles

Fourier-transform infrared (FTIR) spectroscopy was used to analyze the chemical functionalities in the structure of nanoparticle formulations. FTIR spectra of the nanoparticles were obtained from 4000 to 500 cm^−1^ with an average resolution of 4 cm^−1^ (IRSpirit spectrometer, Shimadzu Corp, Kyoto, Japan). The surface morphology and shape of the nanoparticles were investigated by using scanning electron microscopy (SEM). The powder samples were mounted on aluminum stubs, and the nanoparticles were recorded by using the FEI Quanta 650 FEG SEM device (Kyoto, Japan). The sizes of the nanoparticles were measured by using the Zetasizer (Malvern Nano ZS) (Malvern, UK) device, applying the dynamic light scattering (DLS) technique. Average diameter (Z-average) and the polydispersity index (PDI) of the nanoparticles were measured and compared. The encapsulation efficiency (EE, %) and the loading capacity (LC, %) were determined by analyzing the filtrate using UV-Visible Spectrophotometry (Shimadzu 2100S) using the predetermined calibration curves. The EE and the LC were then calculated using Equations and which are given below.

EE (%) = Weight of extract in the NPs/Weight of the extract used × 100

LC (%) = Weight of extract in the NPs/Weight of the NPs × 100

In vitro release profile of extract-loaded nanoparticles from the nanoparticle formulations was investigated spectrophotometrically (Shimadzu 2100S) using dialysis bags in PBS (phosphate-buffered saline, pH 7.4) medium. In total, 25 mg of each nanoformulation and 50 mL of PBS were used for the release experiments. Release experiments were conducted in a thermostatic shaking water bath at 37 °C. Samples were taken at predetermined time intervals (at 1 h, 2 h, 3 h, 4 h, 5 h, 6 h, 8 h, 10 h, 12 h and 24 h) and the concentrations of the samples were calculated by spectrophotometric method using the calibration curves prepared initially [29,54].

### 4.10. Statistical Analysis

All experiments were conducted in triplicate, and the results are presented as mean ± standard deviation (SD). Statistical analysis was performed using GraphPad Prism (version 5.0). Comparisons among multiple groups were carried out using one-way analysis of variance (ANOVA) followed by Tukey’s post hoc test. IC_50_ values were calculated by nonlinear regression analysis. Differences were considered statistically significant at *p* < 0.05.

## Figures and Tables

**Table 1:** Antimicrobial activity of *Onosma alboroseum* subsp. *alboroseum* var. *alboroseum* extracts and extract-loaded nanoparticles.

Microorganisms										
	PE		CE		ME		ME-NP		EF-NP	
	ZD (mm)	MIC/MBC-MFC (mg/mL)	ZD (mm)	MIC/MBC-MFC (mg/mL)	ZD (mm)	MIC/MBC-MFC (mg/mL)	ZD (mm)	MIC/MBC-MFC (mg/mL)	ZD (mm)	MIC/MBC-MFC (mg/mL)
*Escherichia coli* ATCC 25922	0	>30/>30	0	>30/>30	14.24 ± 0.12	3.75/7.5	0	>25/>25	0	>25/>25
*P. aeruginosa* ATCC 27853	0	>30/>30	0	>30/>30	13.17 ± 0.14	7.5/15	0	6.25/25	0	>25/>25
*P. mirabilis* ATCC 14153	0	>30/>30	0	>30/>30	18.07 ± 0.11	7.5/15	0	12.5/>25	0	>25/>25
*A. baumannii* ATCC 19606	11.79 ± 0.11	3.75/7.5	13.3 ± 0.24	1.88/15	18.31 ± 0.13	3.75/30	0	3.13/25	0	>25/>25
*K. pneumoniae* ATCC 4352	0	>30/>30	0	>30/>30	13.95 ± 0.13	7.5/15	0	6.25/25	0	>25/>25
*S. aureus* ATCC 25923	0	>30/>30	0	>30/>30	16.88 ± 0.22	0.94/15	0	>25/>25	0	>25/>25
*S. aureus* ATCC 43300	0	7.5/15	0	7.5/30	21.09 ± 0.31	3.75/15	0	6.25/25	0	>25/>25
*S. epidermidis* ATCC 12228	8.17 ± 0.23	0.94/15	7.66 ± 0.09	0.94/15	12.6 ± 0.24	0.94/15	0	6.25/25	0	>25/>25
*E. faecalis* ATCC 29212	0	>30/>30	0	>30/>30	0	>30/>30	0	>25/>25	0	>25/>25
*C. albicans* ATCC 90028	12.46 ± 0.17	1.88/15 *	12.49 ± 0.14	1.88/15 *	12.74 ± 0.21	1.88/7.5 *	0	3.13/6.25 *	0	>25/>25 *

PE: Petroleum ether extract, CE: Chloroform extract, ME: Methanol extract, ME-NP: Methanol extract-loaded nanoparticles, EF-NP: Extract-free nanoparticles, ZD: Inhibition zone diameter, MIC: Minimal inhibitory concentration, MBC: Minimal bactericidal concentration, MFC: Minimal fungicidal concentration. * *p* < 0.05 compared with the positive control.

**Table 2:** Antioxidant activities and total phenolic content of *Onosma alboroseum* subsp. *alboroseum* var. *alboroseum* extracts and the nanoparticle formulations.

Extracts/Standard	DPPH IC_50_ (mg/mL)	FRAP (mM FeSO_4_ eq./mg Extract)	CUPRAC (mM Trolox eq./mg Extract)	TPC (mg GAE/mg Extract)
Petroleum ether	NA	0.596 ± 0.001 *	0.546 ± 0.024 *	NA
Chloroform	0.987 ± 0.593 *	0.795 ± 0.081 *	1.088 ± 0.050 *	0.014 ± 0.002
Methanol	0.036 ± 0.005 *	2.256 ± 0.072 *	2.595 ± 0.178 *	0.080 ± 0.004
Methanol extract-loaded nanoparticles	1.292 ± 0.649 *	0.145 ± 0.006 *	0.102 ± 0.004 *	NA
Empty nanoparticles	NA	0.023 ± 0.005 *	NA	NA
Ascorbic acid	0.004 ± 0.001		5.683 ± 0.337	
BHA		5.800 ± 0.104		

Values are means of triplicate determination (*n* = 3) ± standard deviation. * *p* < 0.05 compared with the positive control. DPPH: 2,2-diphenyl-1-picrylhydrazyl; GAE: gallic acid equivalent, NA: no activity.

**Table 3:** Acetylcholinesterase and urease inhibitory activities of *Onosma alboroseum* subsp. *alboroseum* var. *alboroseum* extracts and the nanoparticle formulations.

Extracts/Standards	Acetylcholinesterase Inhibition (%) (100 µg/mL)	Urease Inhibition (%) (25 µg/mL)
Petroleum ether	13.108 ± 1.201 *	7.026 ± 0.074 *
Chloroform	8.405 ± 2.250 *	9.123 ± 1.053 *
Methanol	16.109 ± 1.231 *	10.537 ± 1.503 *
Methanol extract-loaded nanoparticles	28.032 ± 2.209 *	12.109 ± 2.132 *
Empty nanoparticles	10.307 ± 1.021 *	8.159 ± 1.105 *
Galantamine	98.240 ± 1.090	
Thiourea		89.050 ± 0.060

Values are means of triplicate determination (*n* = 3) ± standard deviation. * *p* < 0.05 compared with the positive control.

**Table 4:** Tentative identification of compounds detected in the methanol extract of *Onosma alboroseum* subsp. *alboroseum* var. *alboroseum* by LC–ESI–MS analysis.

No.	Rt (min)	Tentative Identification	Molecular Formula	Ion Species	Theoretical *m*/*z*	Experimental *m*/*z*	Diff (Tgt, ppm)	Area%	Compound Class
1	1.272	Piceatannol 3′-O-glucoside	C_20_H_22_O_9_	[M–H]^−^	405.1191	405.1245	4.90	0.310341	Stilbene
2	1.358	4′-O-Methyl-(−)-epicatechin-3′-O-β-glucuronide	C_22_H_22_O_13_	[M–H]^−^	493.1351	493.1254	−3.12	0.005981	Flavan-3-ol
3	2.204	4-Hydroxy-3,5-dimethoxybenzoic acid	C_9_H_10_O_5_	[M+HCOO]^−^	257.0778	257.0779	−0.92	0.886701	Phenolic acid
4	2.576	Succinic acid	C_4_H_6_O_4_	[M–H]^−^	117.0193	117.0193	−0.03	5.467874	Organic acid
5	6.883	Catechin-3-O-(1-hydroxy-6-oxo-2-cyclohexene-1-carboxylate)	C_22_H_20_O_9_	[M–H]^−^	427.1035	427.1050	1.26	0.786821	Flavan-3-ol
6	6.697	Protocatechuic acid 4-glucoside	C_13_H_16_O_9_	[M–H]^−^	315.0722	315.0723	1.47	1.024153	Phenolic acid
7	8.904	Apigenin glucuronide-malonylglucoside derivative	C_30_H_24_O_15_	[M–H]^−^	693.1309	693.1285	−3.20	0.19135	Flavone
8	8.907	Cyanidin-3-O-sambubioside	C_26_H_29_O_15_	[M+HCOO]^−^	661.1177	661.1180	0.20	0.402124	Anthocyanin
9	9.047	Dihydroferulic acid-O-glucuronide	C_16_H_20_O_9_	[M–H]^−^	371.0984	371.0985	−0.40	0.193663	Phenolic acid
10	9.213	Hydroxytyrosol-4-O-glucoside	C_14_H_20_O_8_	[M–H]^−^	315.1085	315.1085	−0.02	1.713512	Phenylethanoid
11	9.382	Quercetin glycoside derivative	C_36_H_46_O_20_	[M–H]^−^	797.2510	797.2489	−3.75	0.701626	Flavonol
12	9.408	Kaempferol-3,4′-diglucoside	C_27_H_30_O_16_	[M–H]^−^	549.1250	549.1367	−0.03	0.089735	Flavonol
13	9.491	Cis-p-coumaric acid 4-[apiosyl-(1->2)-glucoside	C_20_H_26_O_12_	[M–H]^−^	457.1351	457.1324	−5.06	0.719737	Phenolic acid
14	9.548	Caffeic acid	C_9_H_8_O_4_	[M–H]^−^	179.0349	179.0350	−0.64	4.721373	Phenolic acid
15	9.647	Benzoyl glucuronide	C_13_H_14_O_8_	[M–H]^−^	297.0615	297.0616	−0.27	1.121813	Phenolic acid
16	10.377	Cinnamoyl glucose	C_15_H_18_O_7_	[M–H]^−^	309.0980	309.0942	−1.72	0.013553	Phenylpropanoid
17	11.924	7-Acetoxy-4-methylcoumarin	C_12_H_10_O_4_	[M-H]^−^	233.0665	233.0667	−0.32	0.671046	Coumarin
18	12.494	Acacetin-7-(4’’’-acetylrutinoside)	C_30_H_34_O_14_	[M–H]^−^	633.1825	633.1796	−4.33	0.807881	Flavone
19	12.816	Apigenin-7-xyloside	C_20_H_20_O_9_	[M+HCOO]^−^	447.0933	447.0930	−0.57	19.89322	Flavone
20	13.580	Rosmarinic acid	C_18_H_16_O_8_	[M–H]^−^	359.0768	359.0772	−0.94	48.89368	Phenolic acid
21	13.619	Chicoric acid	C_22_H_18_O_12_	[M–H]^−^	473.0725	473.0700	−4.23	6.191134	Phenolic acid
22	13.878	Secoisolariciresinol	C_20_H_26_O_6_	[M–H]^−^	361.1655	361.1657	−0.31	0.443396	Lignan
23	21.215	7,4′,5′-Trihydroxy-5,2′-oxido-4-phenylcoumarin	C_15_H_8_O_6_	[M+HCOO]^−^	329.0303	329.0294	−2.79	2.226194	Coumarin
24	31.372	Luteolin sulfate-rutinoside derivative	C_33_H_30_O_18_S	[M–H]^−^	673.1080	673.1116	3.56	2.421236	Flavone

Rt: Retention time. *m*/*z* values correspond to deprotonated molecules ([M–H]^−^) or adduct ions formed in negative ESI mode. Diff (ppm): Mass error expressed in parts per million relative to theoretical *m*/*z*. Area% values represent relative peak areas normalized to the most intense detected feature (base peak = 100%) obtained from the full-scan LC–ESI–QTOF–MS chromatogram. These values provide a semi-quantitative comparison and do not reflect absolute concentrations. Compound identification is tentative and based on accurate mass measurement, isotopic pattern matching, and database comparison.

**Table 5:** Encapsulation efficiency (EE) and loading capacity (LC) of extract-loaded nanoparticles.

Nanoparticle	Encapsulation Efficiency, EE (%)	Loading Capacity, LC (%)
ONP	29.7	6.89

ONP: Methanol extract-loaded nanoparticle.

## Data Availability

The original contributions presented in this study are included in the article/Supplementary Materials. Further inquiries can be directed to the corresponding author.

## Supplementary Materials

The following supporting information can be downloaded at: https://www.mdpi.com/article/10.3390/ph19030451/s1. Figures S1–S24: Representative full-scan mass spectra of selected compounds.
