# Supplementary material for: Biological Activities and LC–QTOF–MS-Based Phytochemical Characterization of Onosma alboroseum Fisch. et Mey. subsp. alboresum var. alboroseum Extracts and Extract-Loaded Nanoparticles
Source: Pharmaceuticals (Basel). 2026 Mar 11;19(3):451. doi: 10.3390/ph19030451 (PMC13029086; doi:10.3390/ph19030451)
Supplement: Supplementary file 1 [file pharmaceuticals-19-00451-s001.zip › pharmaceuticals-4171746-supplementary.pdf]

Article

Biological Activities and LC–QTOF–MS-Based Phytochemical Characterization of *Onosma alboroseum* Fisch. et Mey. subsp. *alboresum* var. *alboroseum* Extracts and Extract-Loaded Nanoparticles

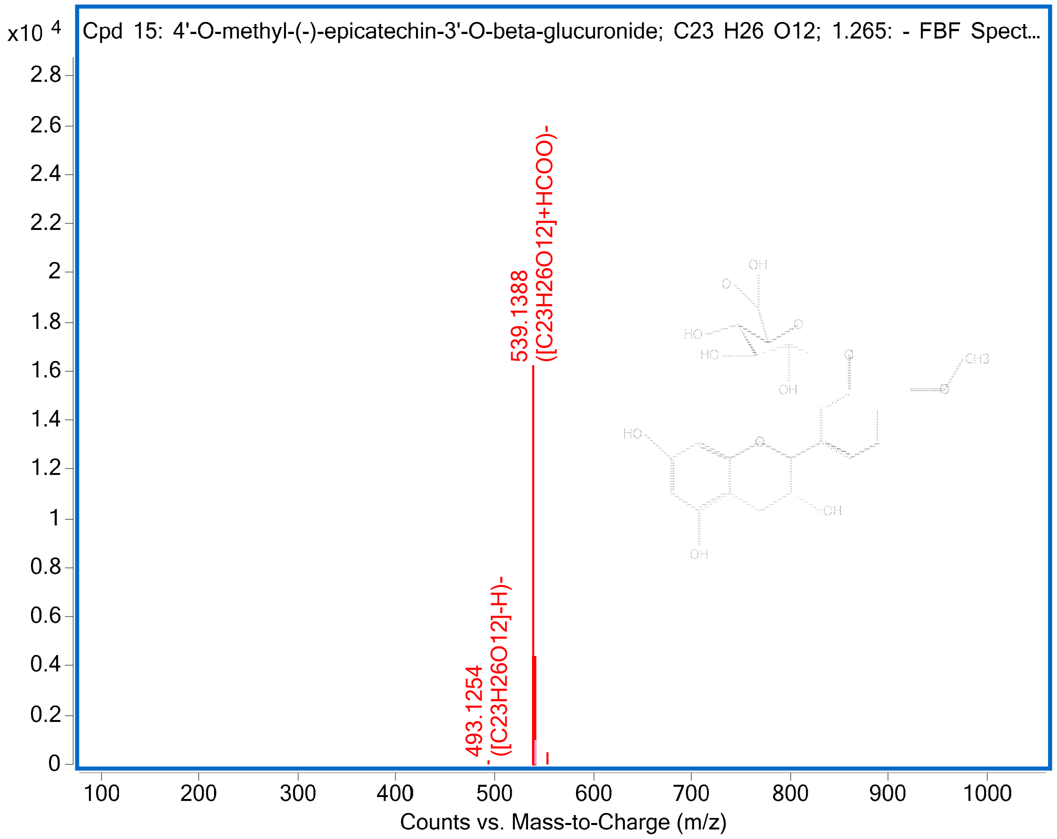

Figure S1. Negative ESI-MS full-scan spectrum of 4′-O-methyl-(−)-epicatechin-3′-O-β-glucuronide.

4′-O-methyl-(−)-epicatechin-3′-O-β-glucuronide was tentatively identified using LC–HRMS in negative ESI mode based on accurate mass measurement, showing a deprotonated molecular ion at m/z 493.12. Full-scan spectra are provided as Supplementary Information.

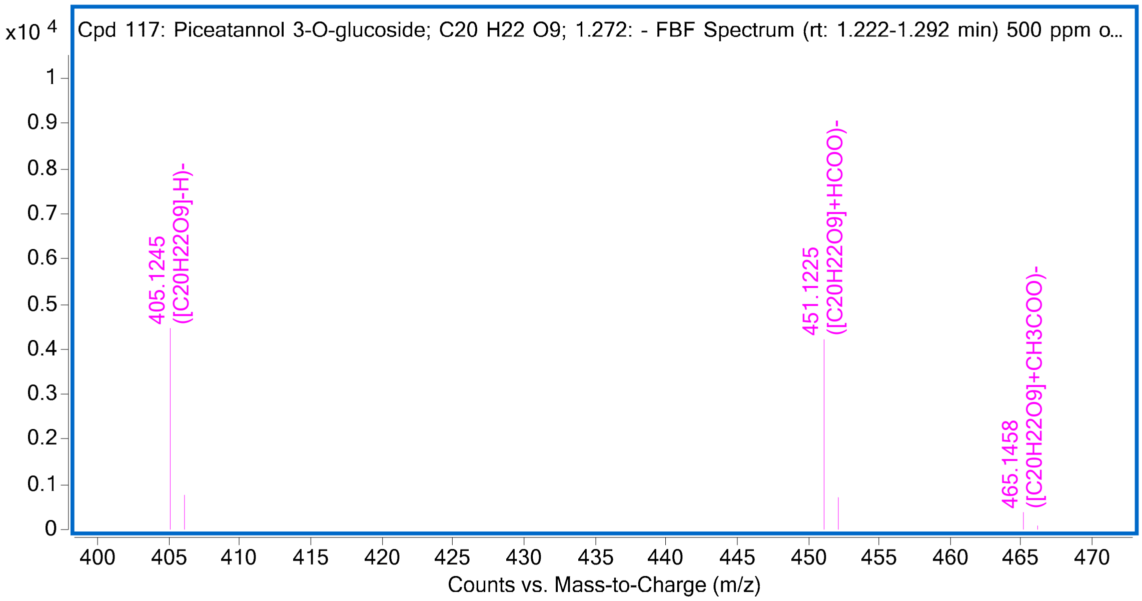

Figure S2. Negative ESI-MS full-scan spectrum of piceatannol-3-O-glucoside.

Piceatannol-3-O-glucoside was tentatively identified in negative ESI mode based on its deprotonated molecular ion at m/z 405.12 $[M-H]^-$, along with characteristic formate and acetate adduct ions at m/z 451.12 and 465.15, respectively. Full-scan spectra are shown in the Supplementary Information.

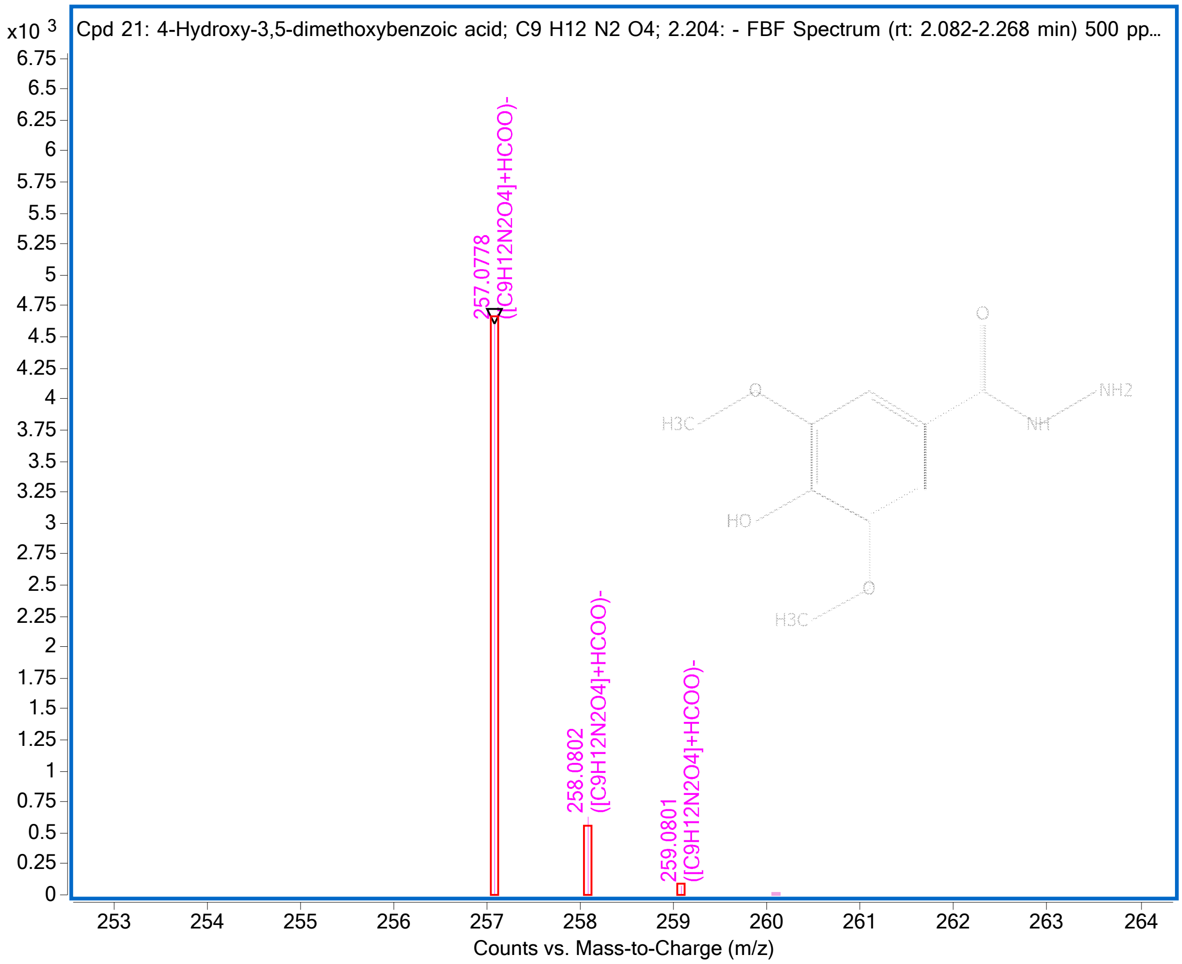

Figure S3. Negative ESI-MS full-scan spectrum of 4-hydroxy-3,5-dimethoxybenzoic acid.

4-Hydroxy-3,5-dimethoxybenzoic acid was detected predominantly as formate adducts or in-source clusters in negative ESI mode during acquisition. Therefore, compound assignments were made conservatively based on accurate mass, retention behavior, and characteristic fragmentation patterns.

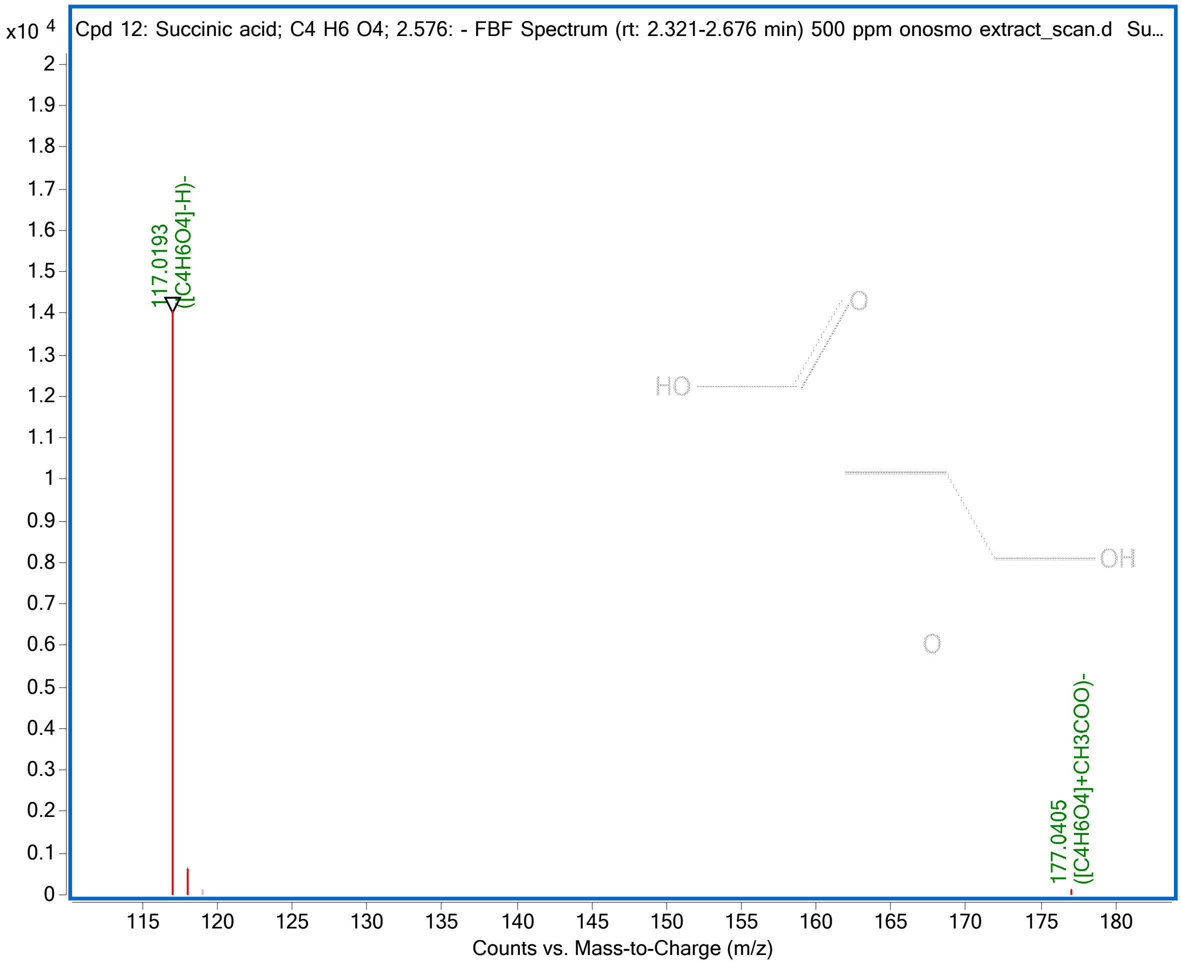

Figure S4. Negative ESI-MS full-scan spectrum of succinic acid.

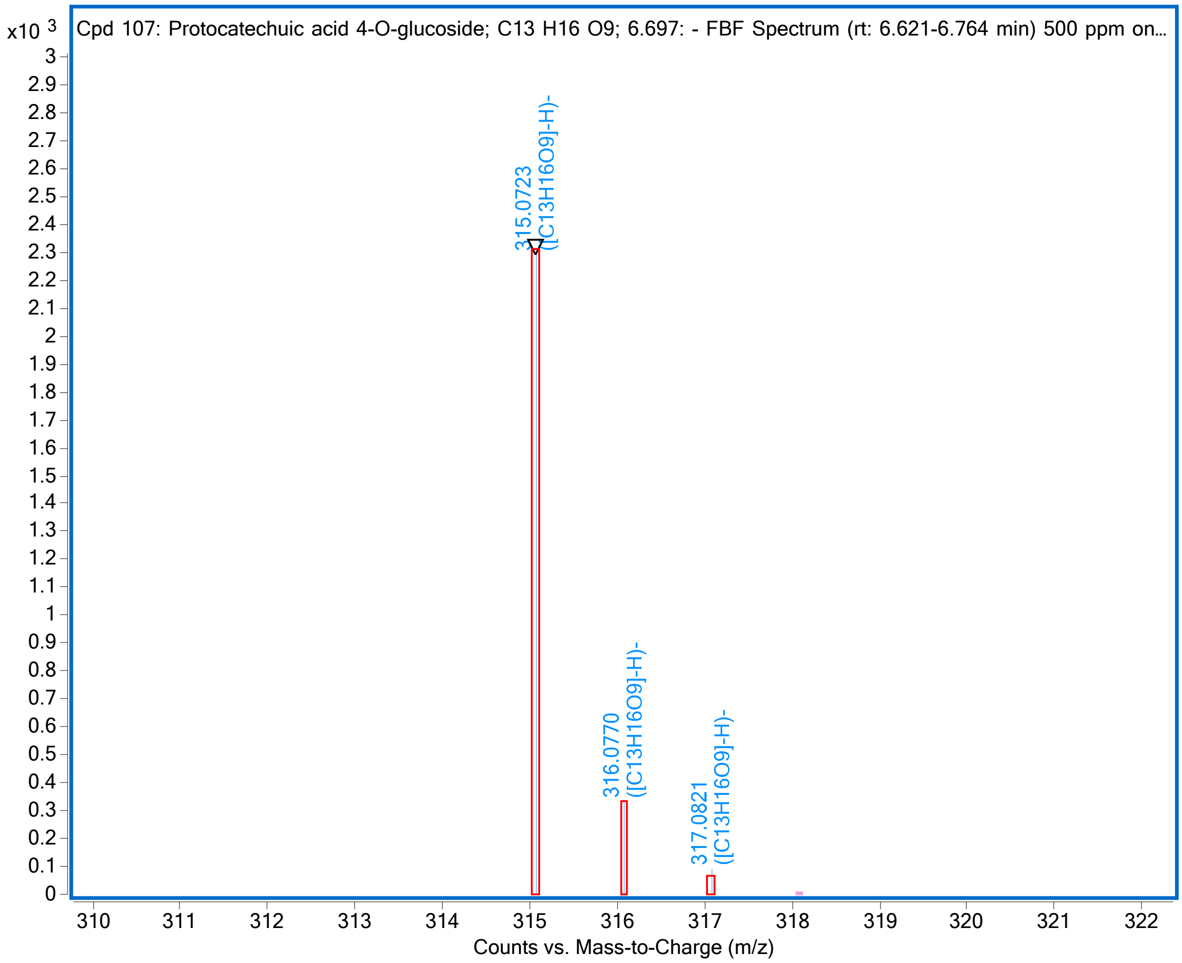

Figure S5. Negative ESI-MS full-scan spectrum of Protocatechuic acid 4-O-glucoside.

Protocatechuic acid 4-O-glucoside was tentatively identified by LC–HRMS in negative ESI mode based on the presence of a dominant deprotonated molecular ion at m/z 315.07 $[M-H]^-$. The corresponding full-scan mass spectrum is provided as Supplementary Information.

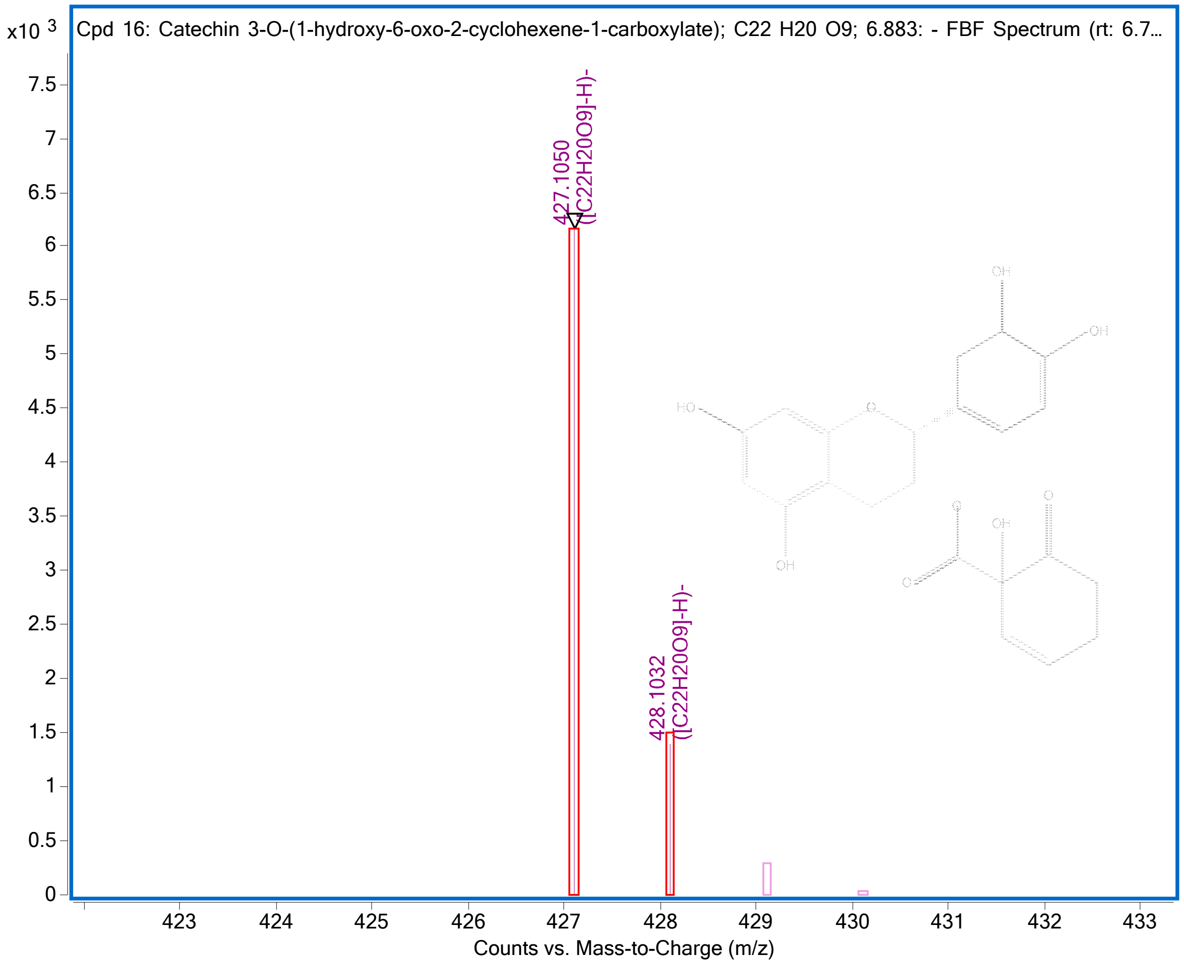

Figure S6. Negative ESI-MS full-scan spectrum of Catechin 3-O-(1-hydroxy-6-oxo-2-cyclohexene-1-carboxylate).

Catechin 3-O-(1-hydroxy-6-oxo-2-cyclohexene-1-carboxylate) was tentatively identified by LC–HRMS in negative ESI mode based on the presence of a dominant deprotonated molecular ion at m/z 427.11 $[M{-}H]^-$. The corresponding full-scan mass spectrum is provided as Supplementary Information.

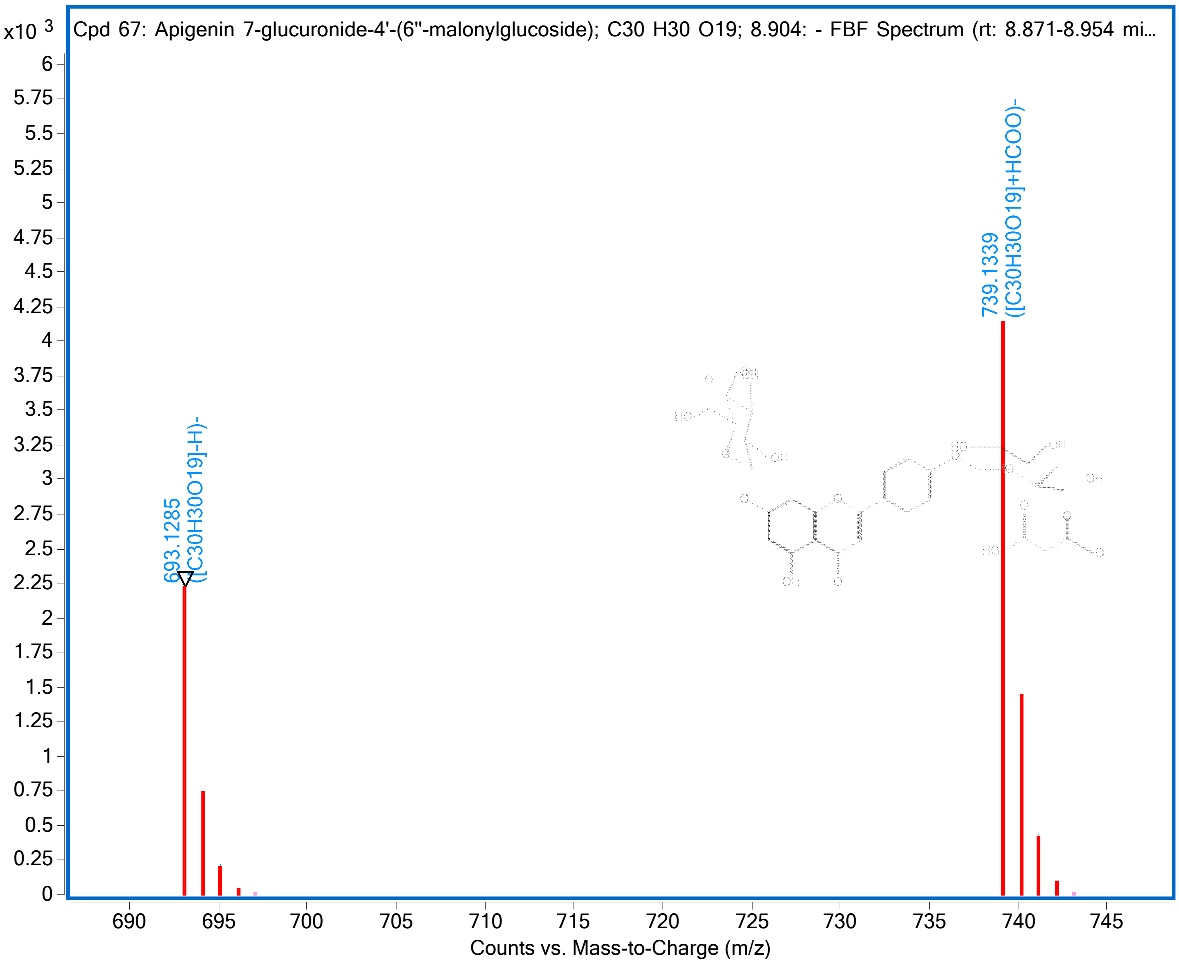

Figure S7. Negative ESI-MS full-scan spectrum of Apigenin 7-glucuronide-4′-(6″-malonylglucoside)

Apigenin 7-glucuronide-4′-(6″-malonylglucoside) was tentatively identified by LC–HRMS in negative ESI mode based on a deprotonated molecular ion at m/z 693.13 [M−H]$^-$, along with a formate adduct ion at m/z 739.13. The corresponding full-scan mass spectrum is provided as Supplementary Information.

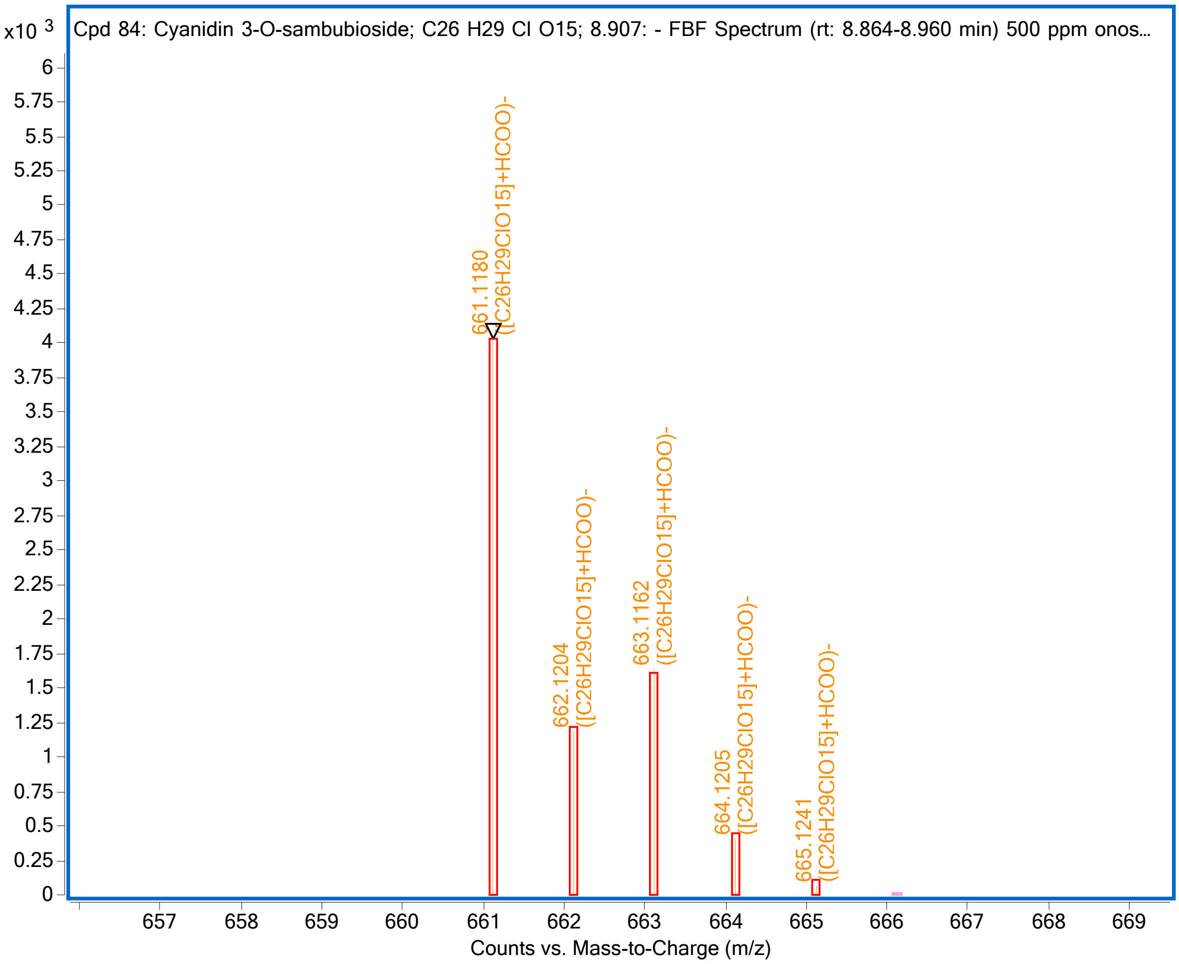

Figure S8. Negative ESI-MS full-scan spectrum of Cyanidin 3-O-sambubioside

Cyanidin 3-O-sambubioside was tentatively identified by LC–HRMS in negative ESI mode based on a deprotonated molecular ion at m/z 611.18 $[M-H]^-$, together with formate and acetate adduct ions at m/z 657.18 and 669.18, respectively. The corresponding full-scan mass spectrum is provided as Supplementary Information.

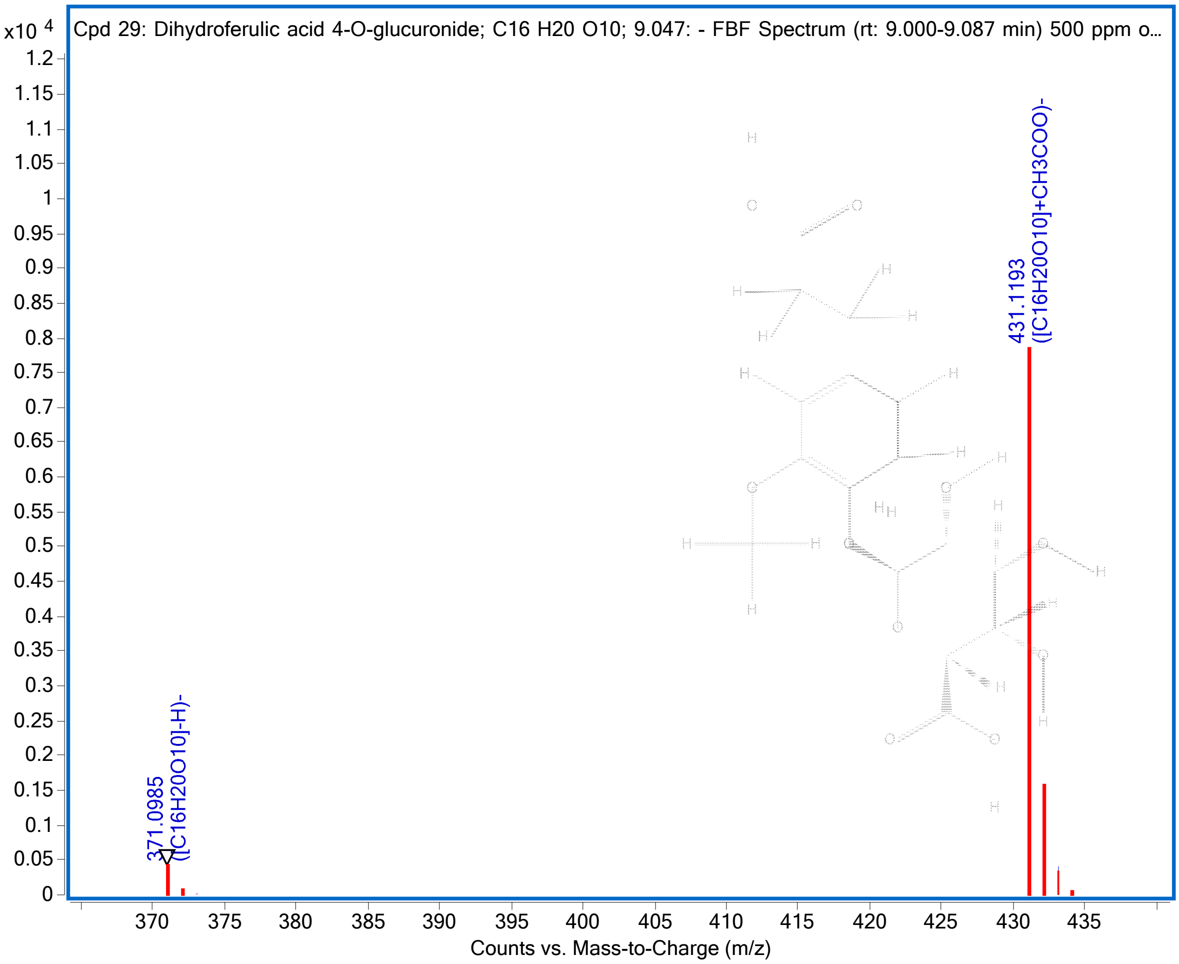

Figure S9. Negative ESI-MS full-scan spectrum of Dihydroferulic acid 4-O-glucuronide

Dihydroferulic acid 4-O-glucuronide was tentatively identified by LC–HRMS in negative ESI mode based on the presence of a dominant formate adduct ion at m/z 431.11 $[M+HCOO]^{-}$. The corresponding full-scan mass spectrum is provided as Supplementary Information.

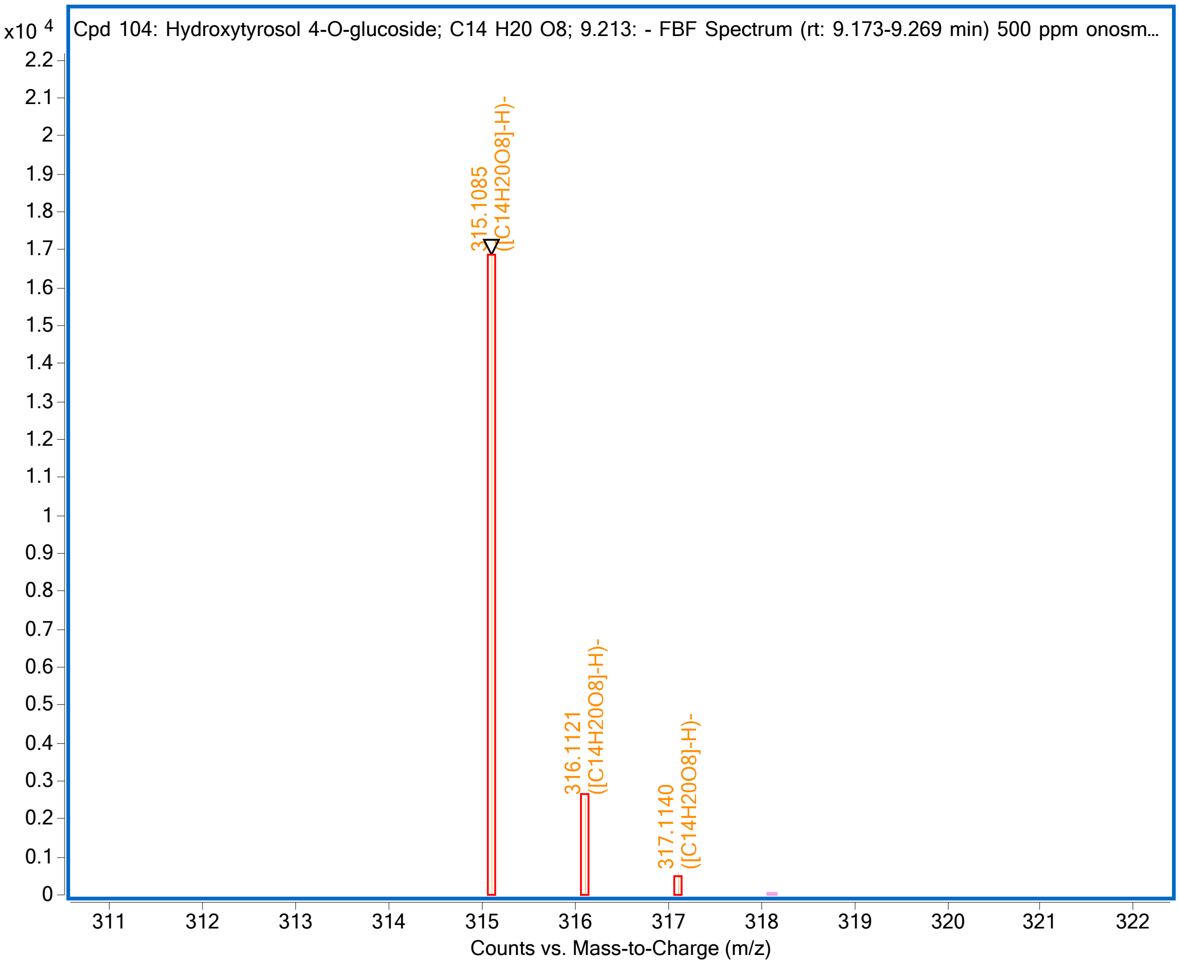

Figure S10. Negative ESI-MS full-scan spectrum of Hydroxytyrosol 4-O-glucoside

Hydroxytyrosol 4-O-glucoside was tentatively identified by LC–HRMS in negative ESI mode based on the presence of a dominant deprotonated molecular ion at m/z 315.11 $[M-H]^-$. The corresponding full-scan mass spectrum is provided as Supplementary Information.

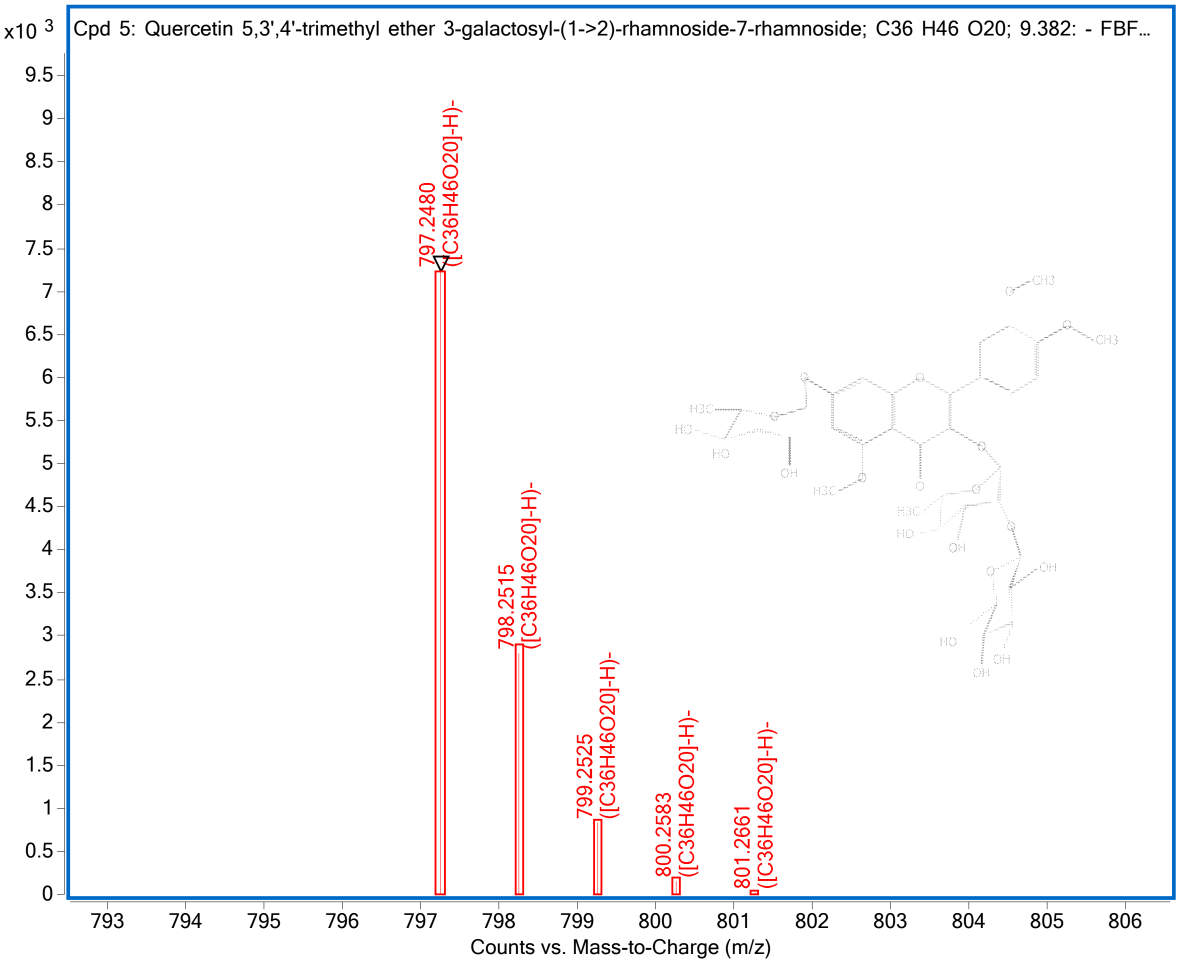

Figure S11. Negative ESI-MS full-scan spectrum of Quercetin 5,3',4'-trimethyl ether 3-galactosyl-(1->2)-rhamnoside-7-rhamnoside.

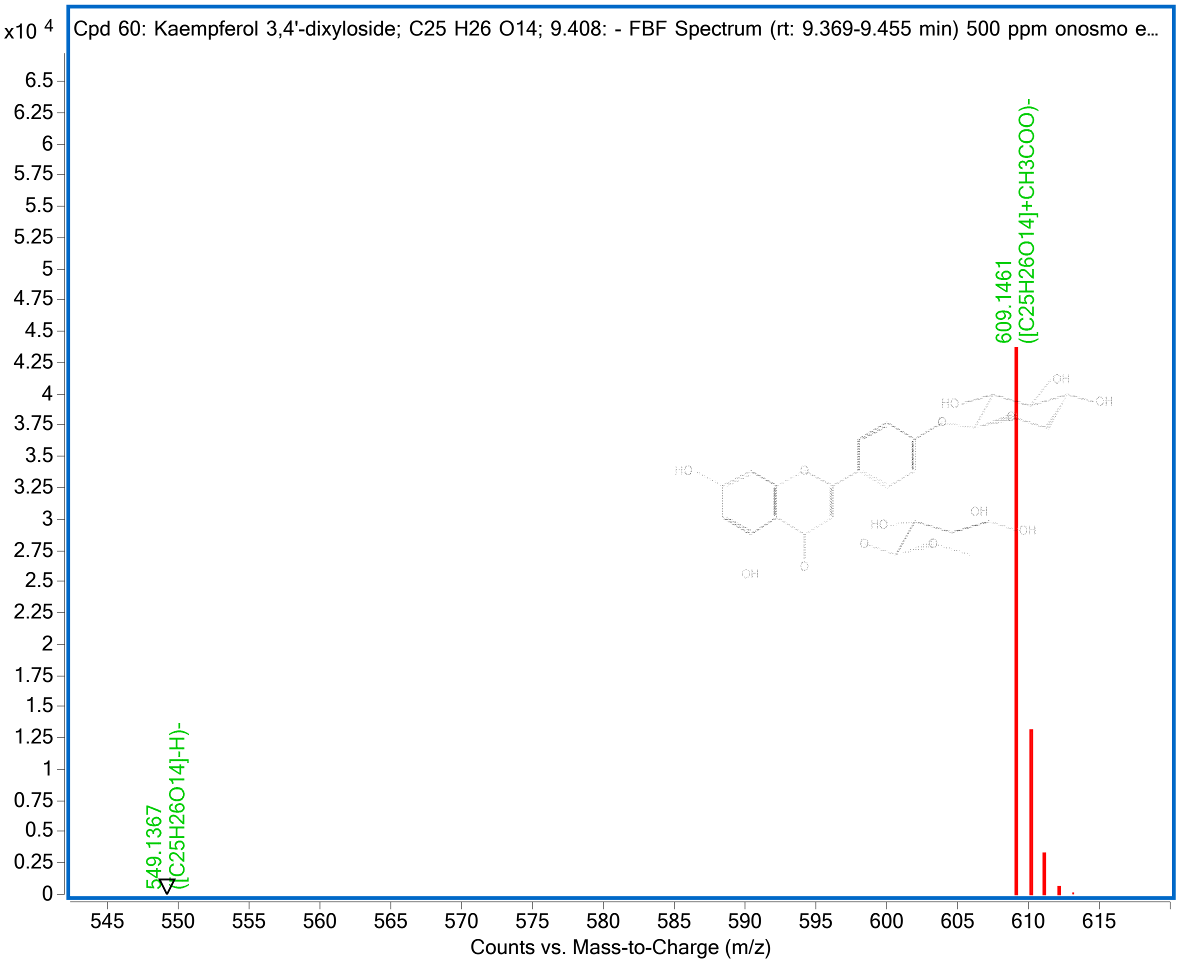

Figure S12. Negative ESI-MS full-scan spectrum of Kaempferol-3,4′-diglucoside.

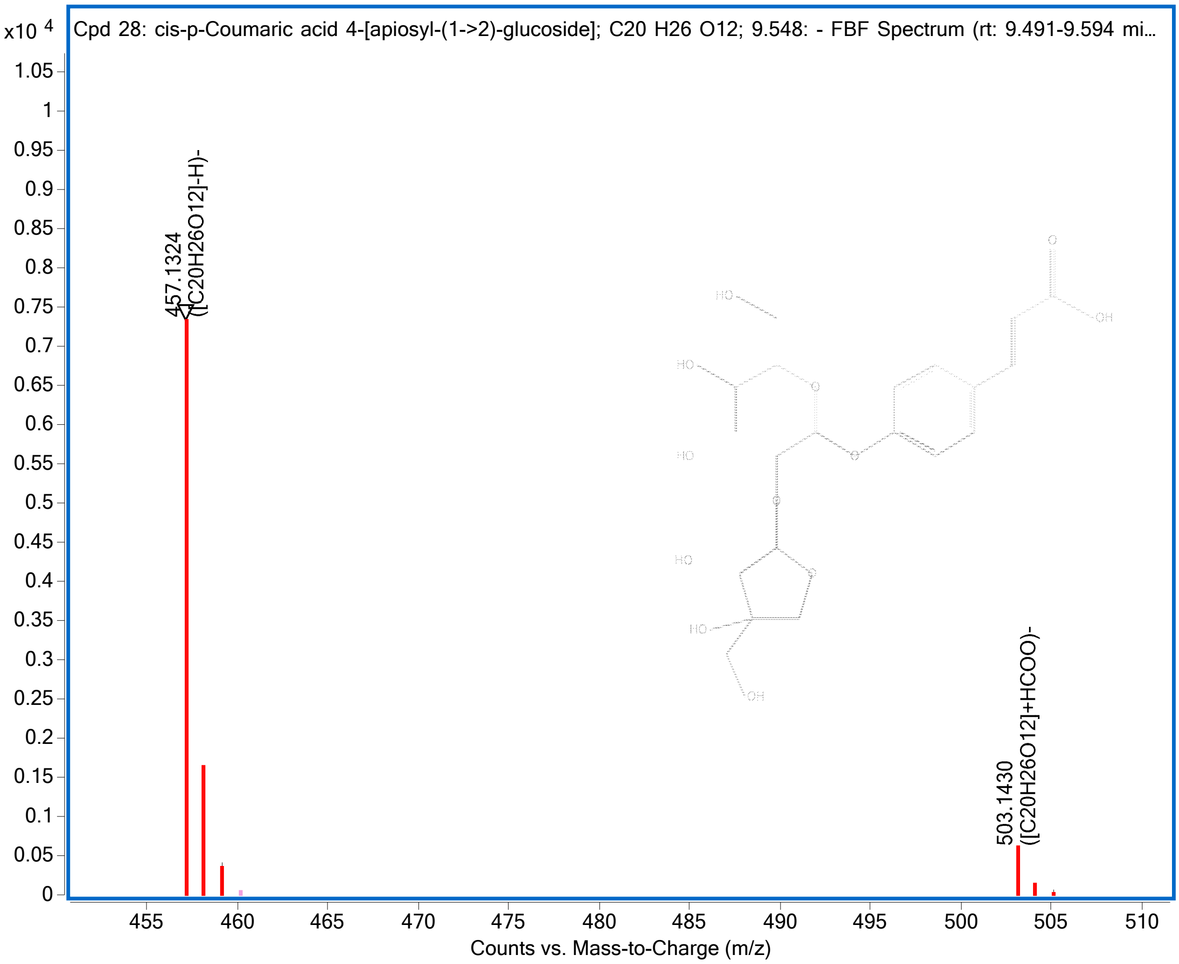

Figure S13. Negative ESI-MS full-scan spectrum of Cis-p-coumaric acid 4-[apiosyl-(1->2)-glucoside.

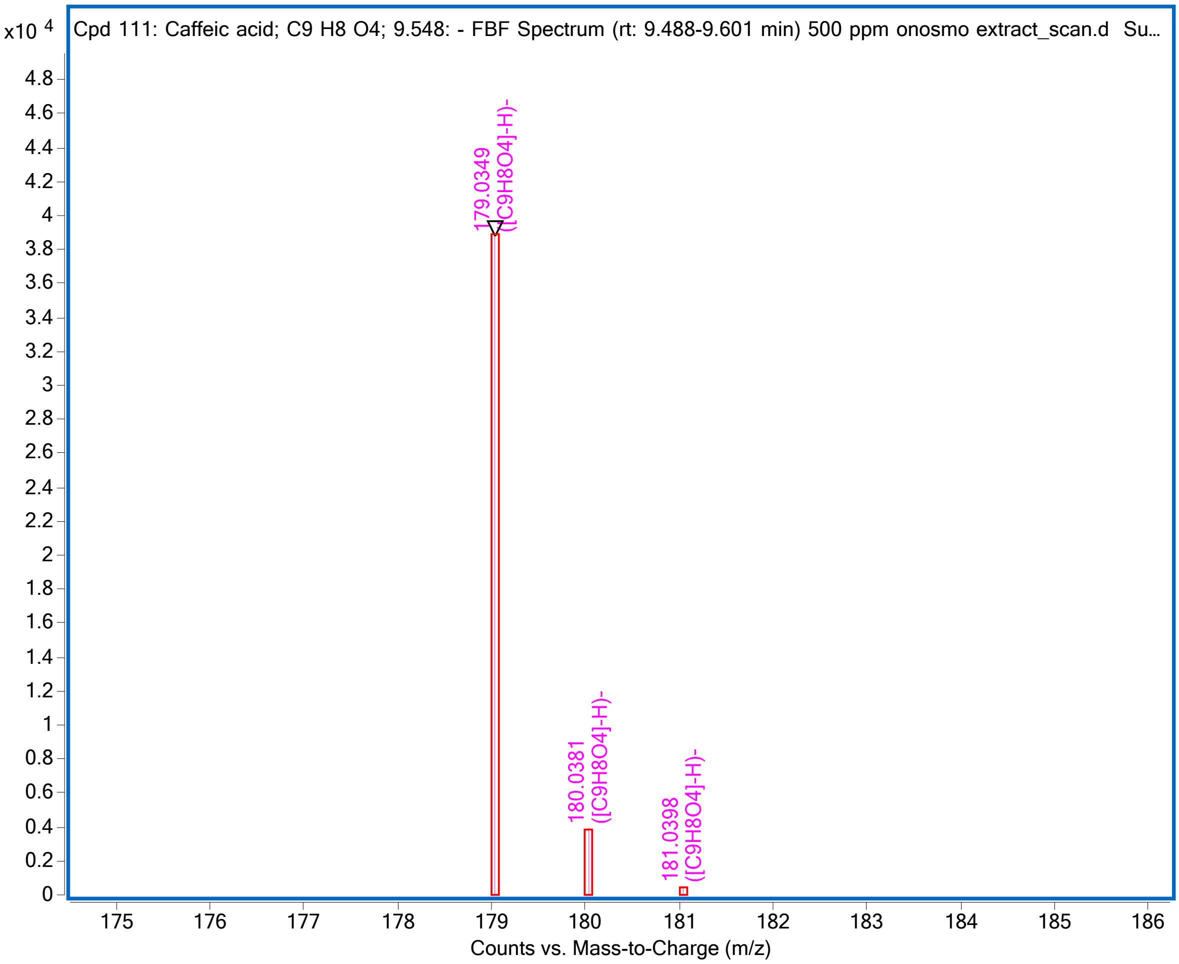

Figure S14. Negative ESI-MS full-scan spectrum of caffeic acid.

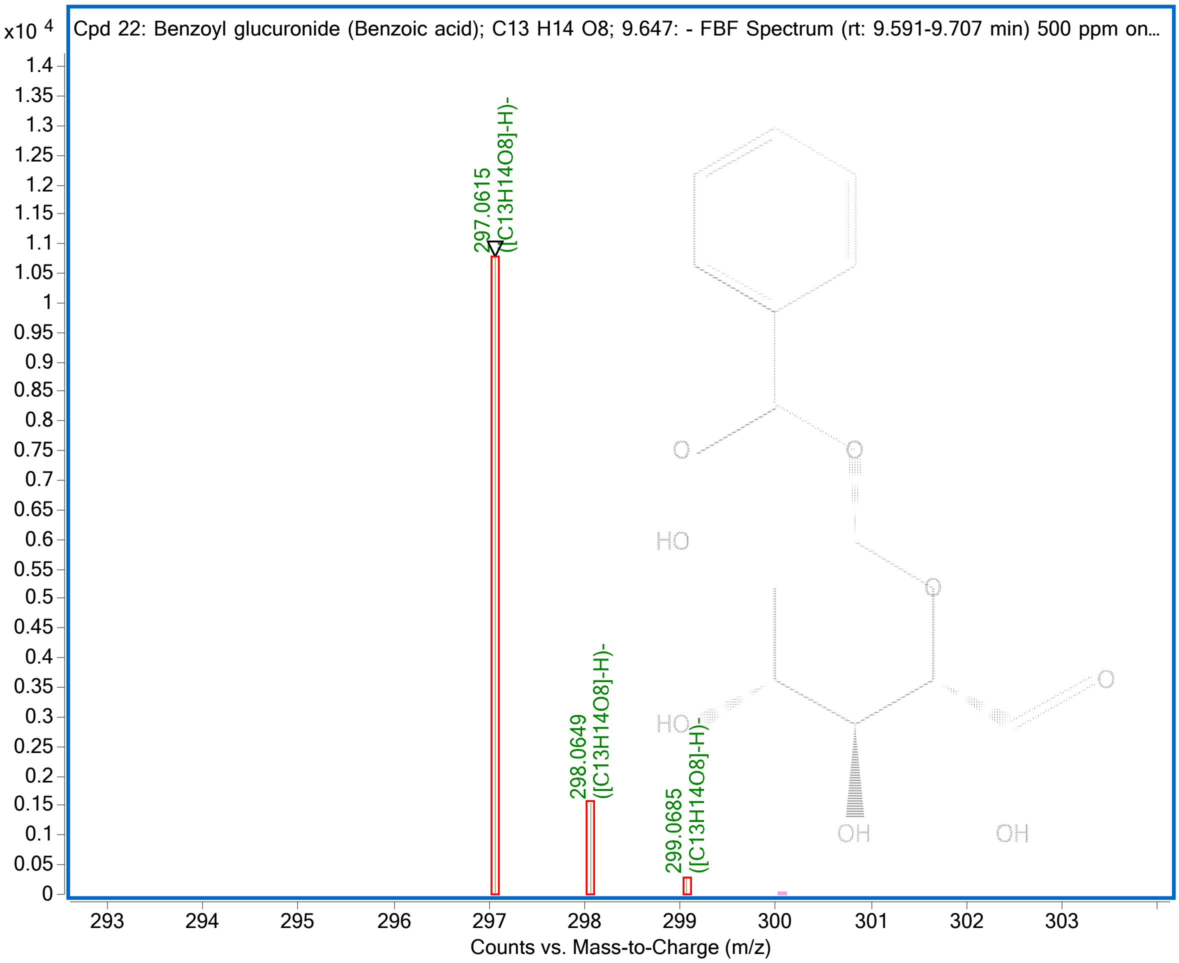

Figure S15. Negative ESI-MS full-scan spectrum of benzoic acid.

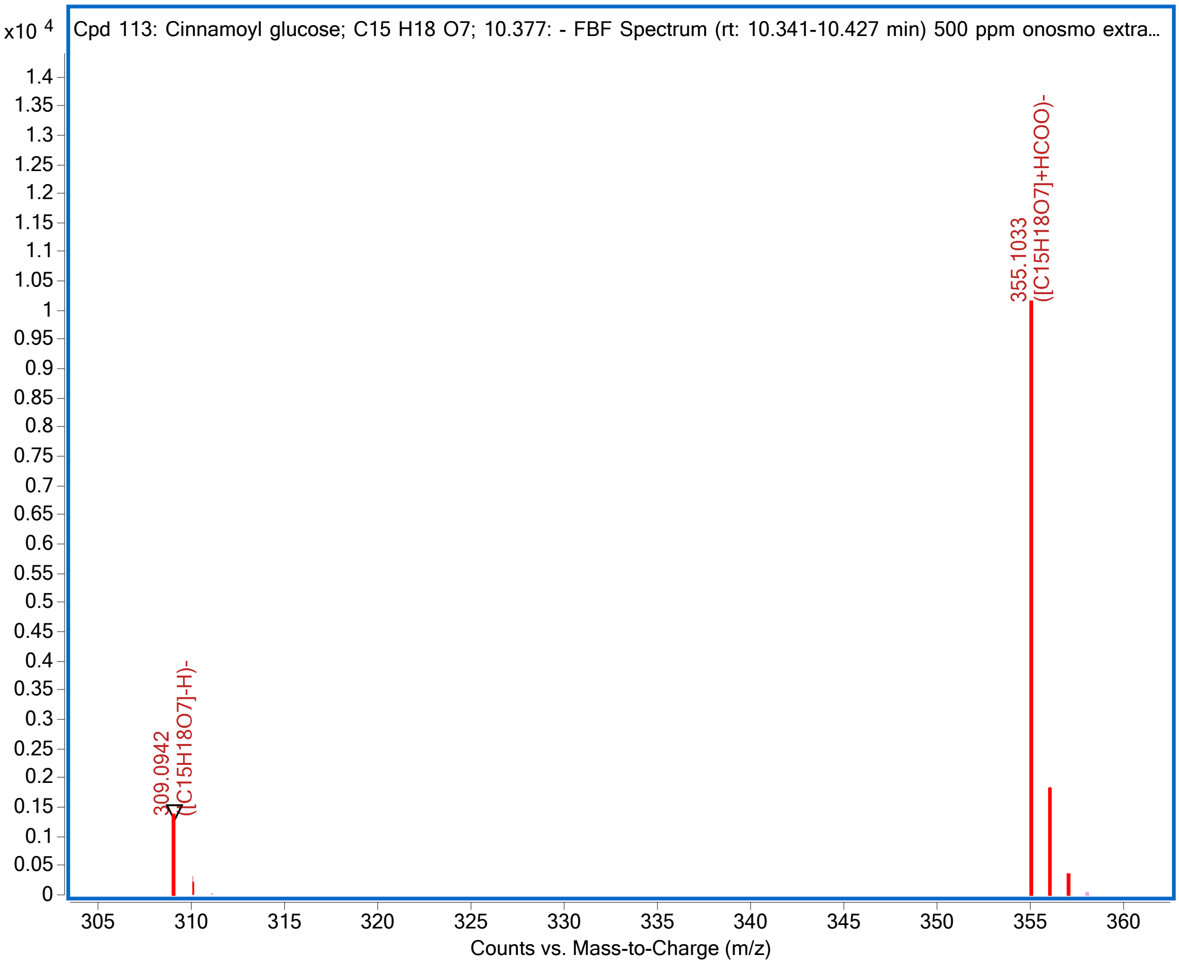

Figure S16. Negative ESI-MS full-scan spectrum of Cinnamoyl glucose.

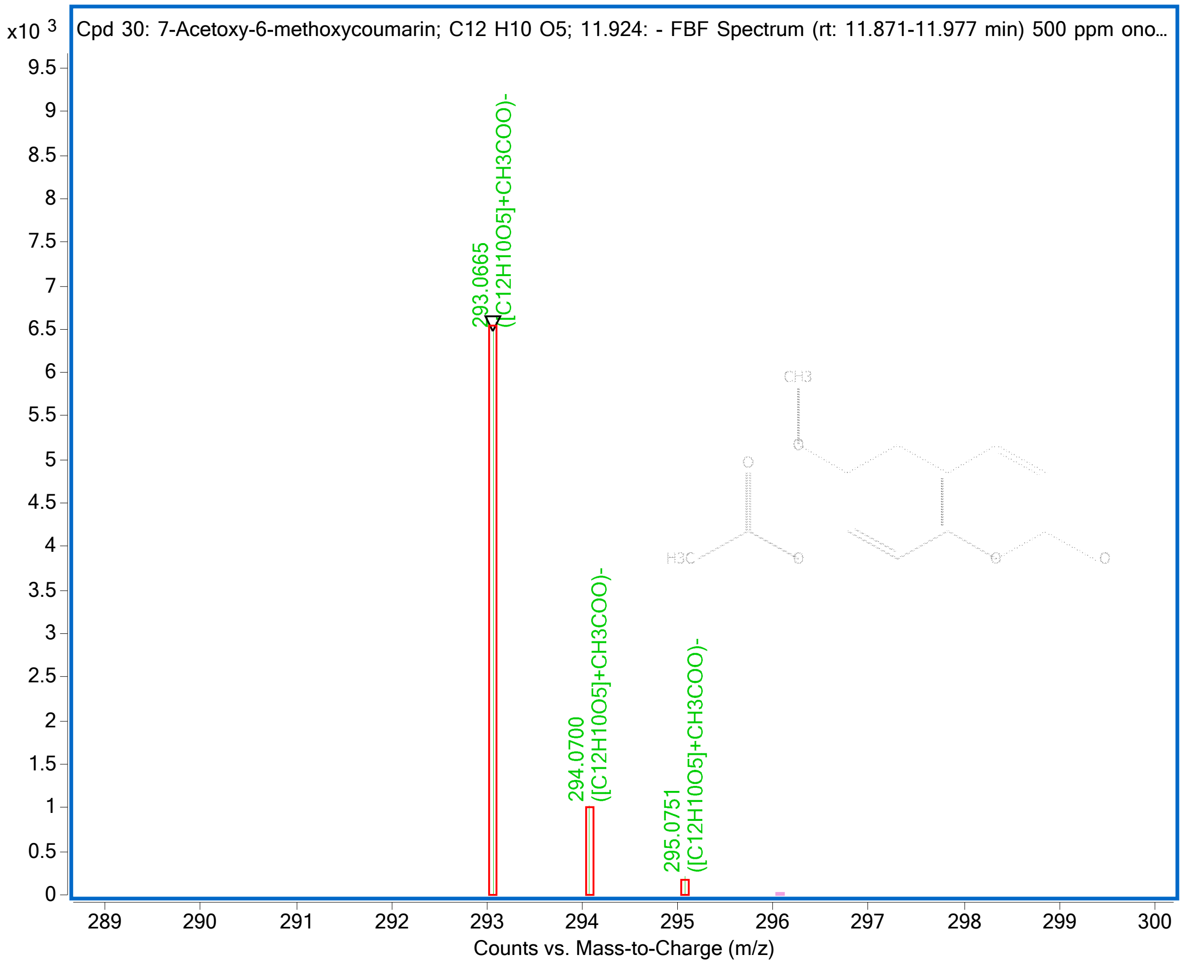

Figure S17. Negative ESI-MS full-scan spectrum of 7-Acetoxy-6-methoxycoumarin showing the detected molecular-related ion at m/z 293.

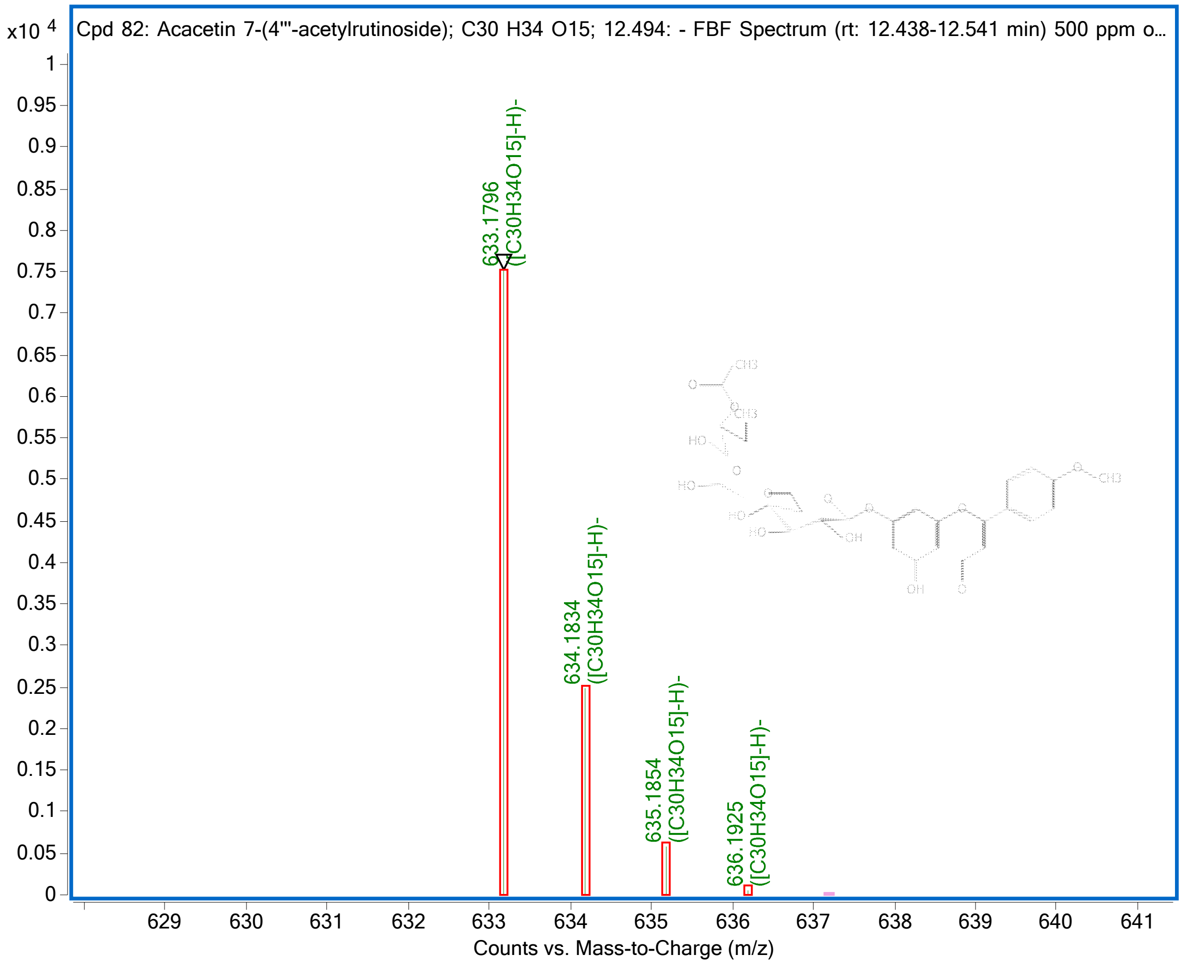

Figure S18. Negative ESI-MS full-scan spectrum of Acacetin-7-(4```-acetylrutinoside)

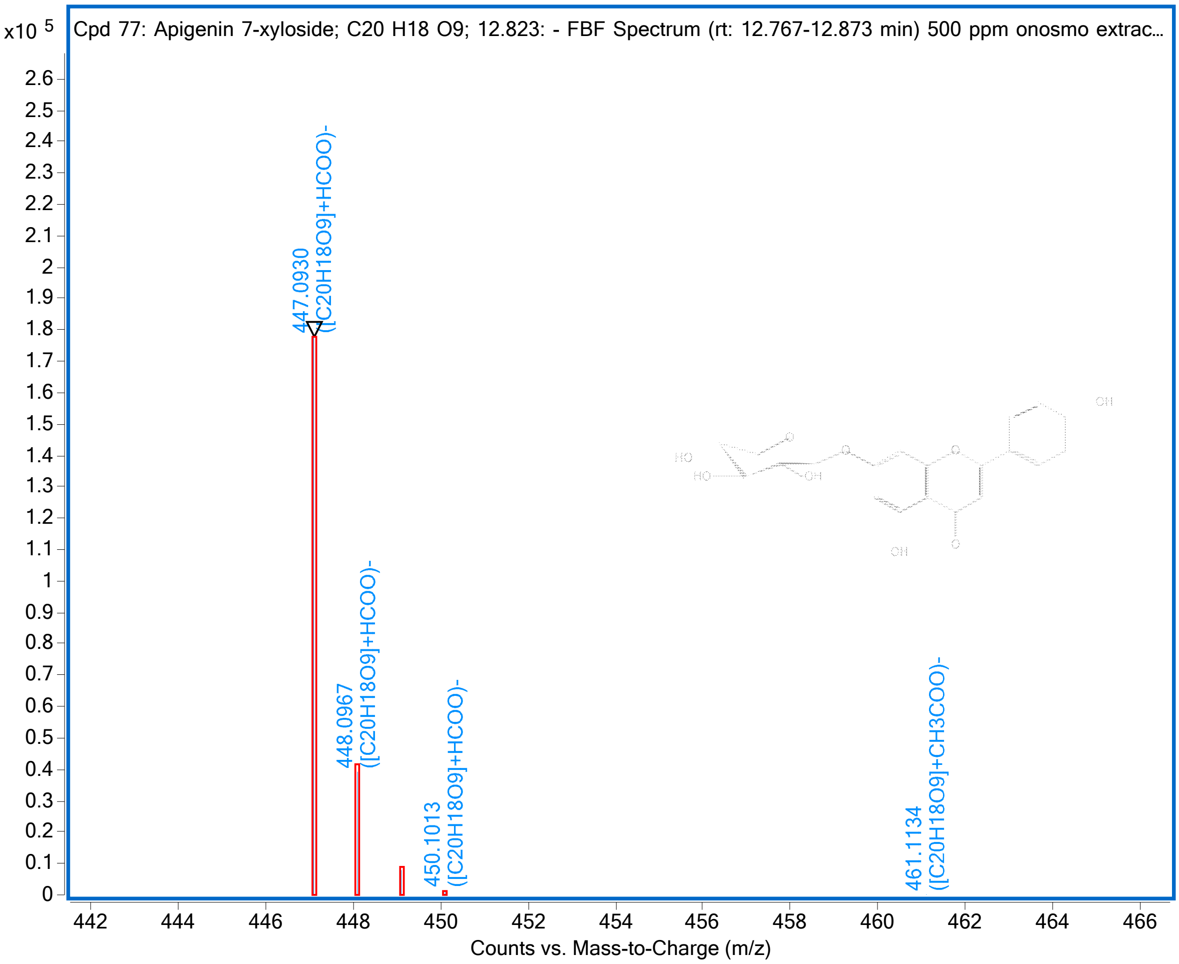

Figure S19. Negative ESI-MS full-scan spectrum of Apigenin-7-O-xyloside showing the formate adduct ion [M + HCOO]⁻ at m/z 447.0930 formed in the presence of formic acid in the mobile phase.

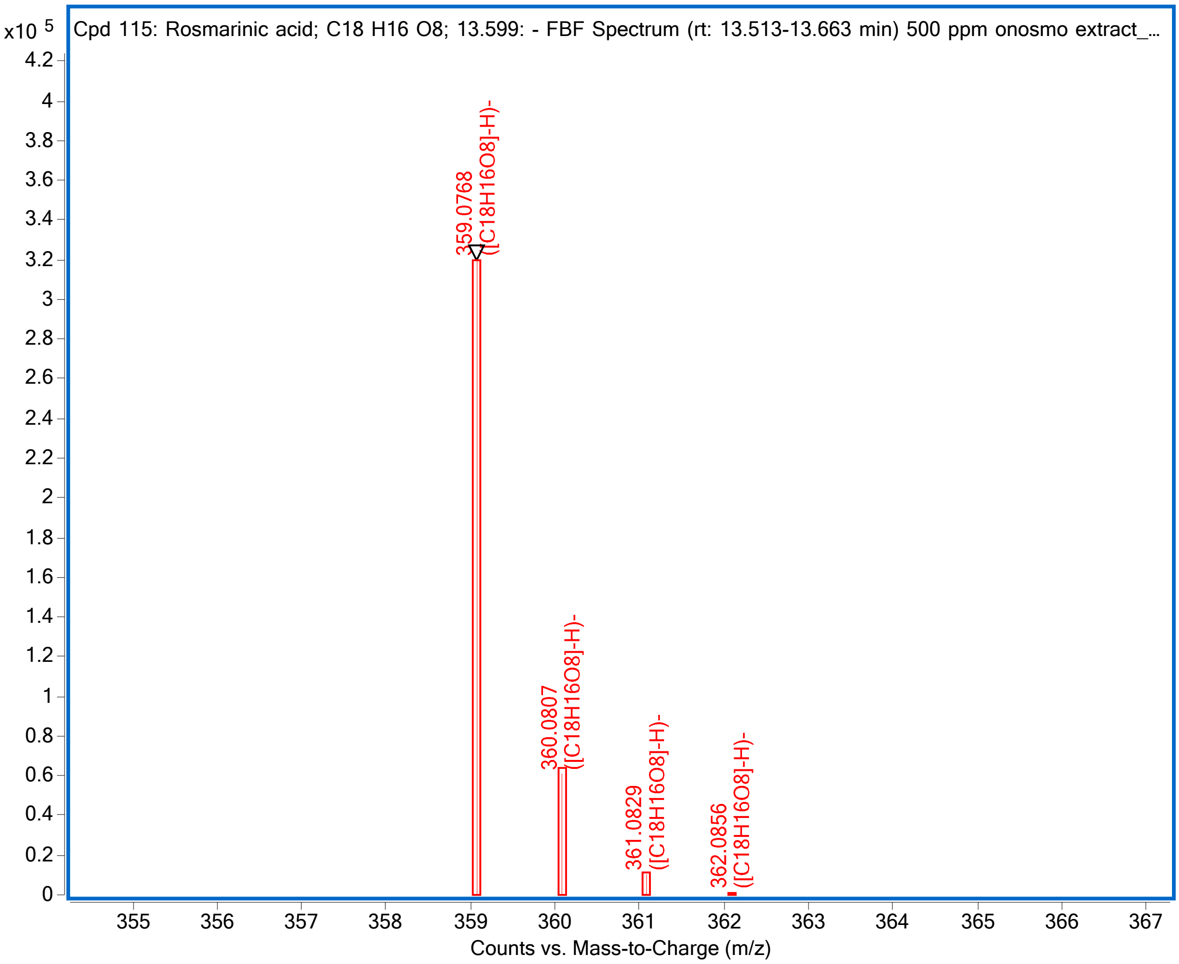

Figure S20. Negative ESI-MS full-scan spectrum of rosmarinic acid

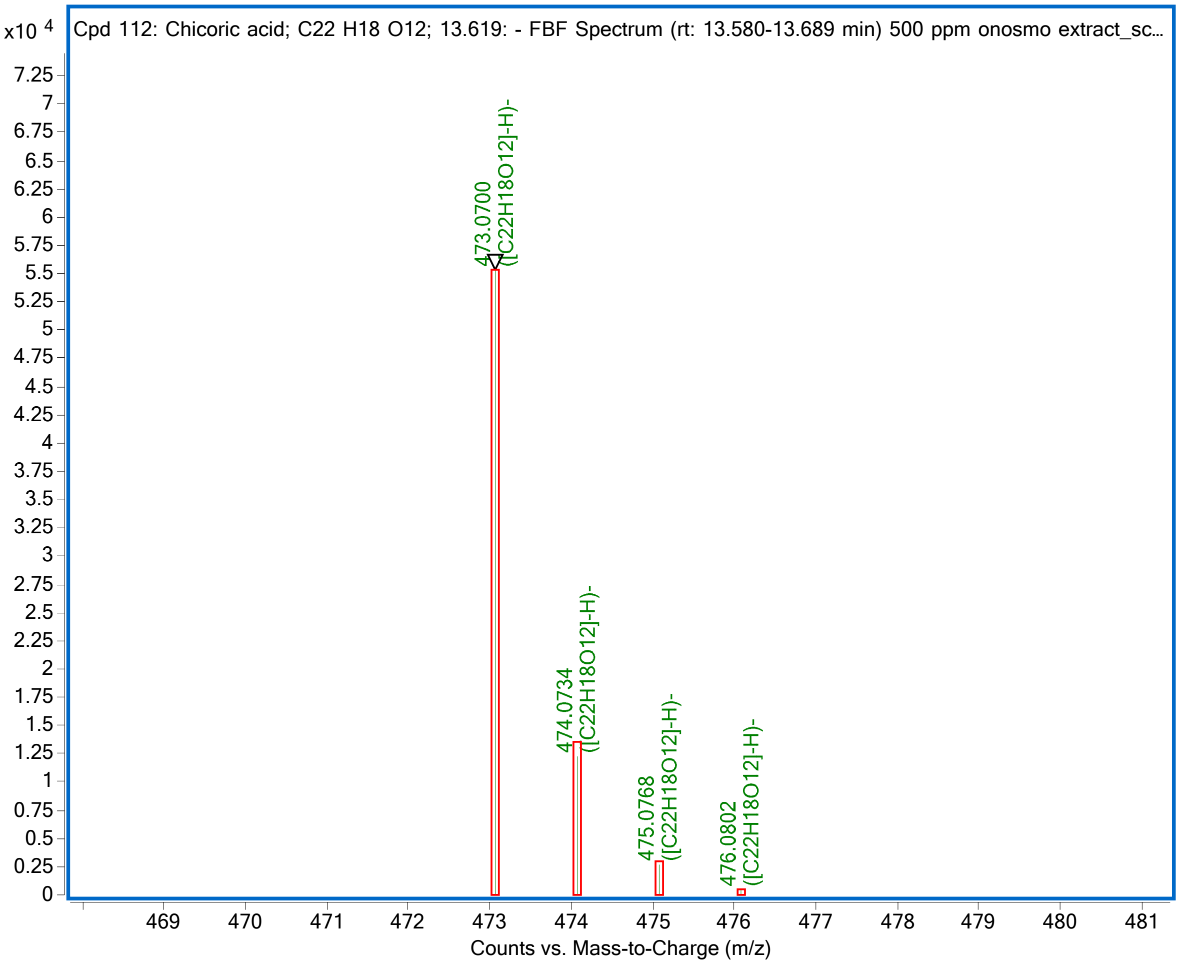

Figure S21. Negative ESI-MS full-scan spectrum of Chicoric acid

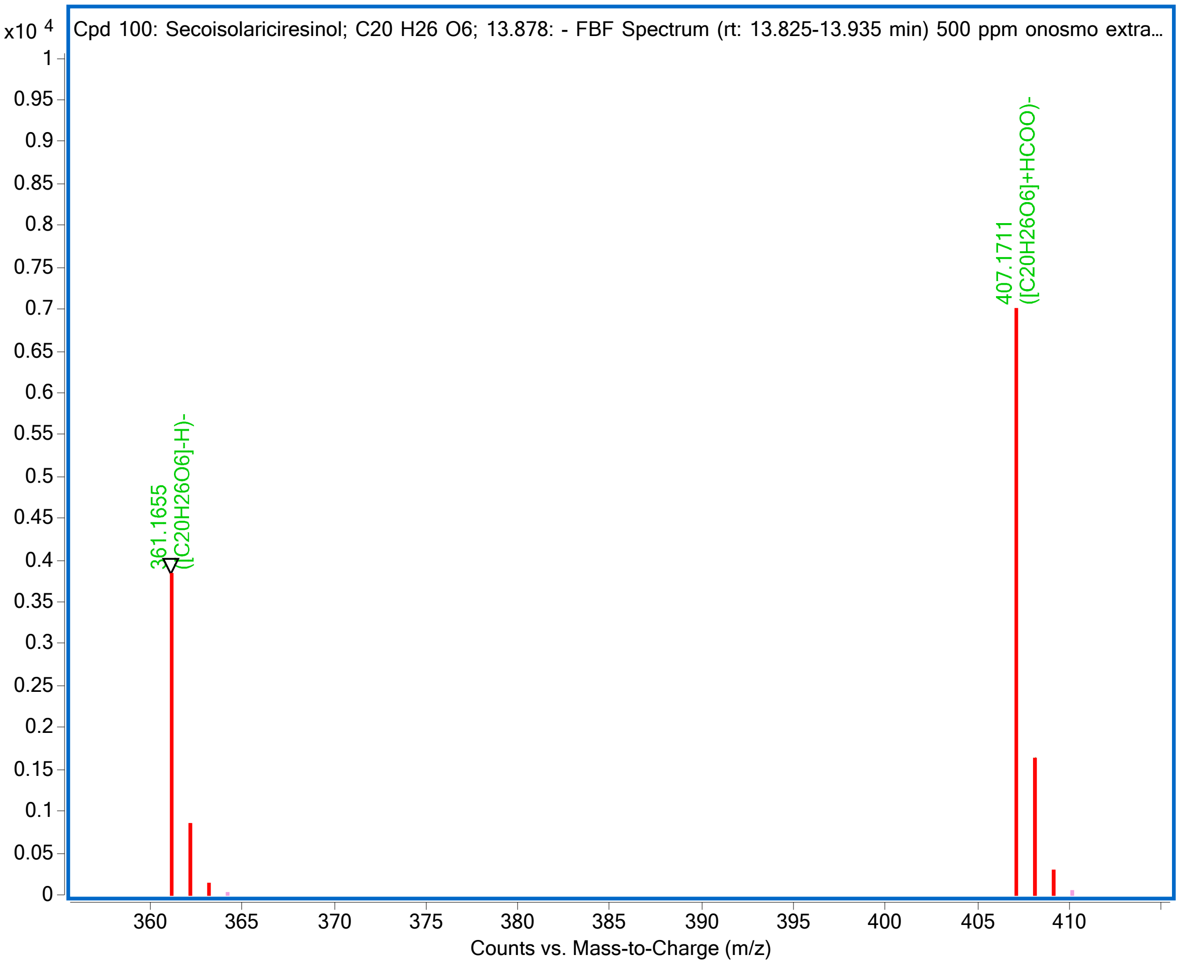

Figure S22. Negative ESI-MS full-scan spectrum of Secoisolariciresinol

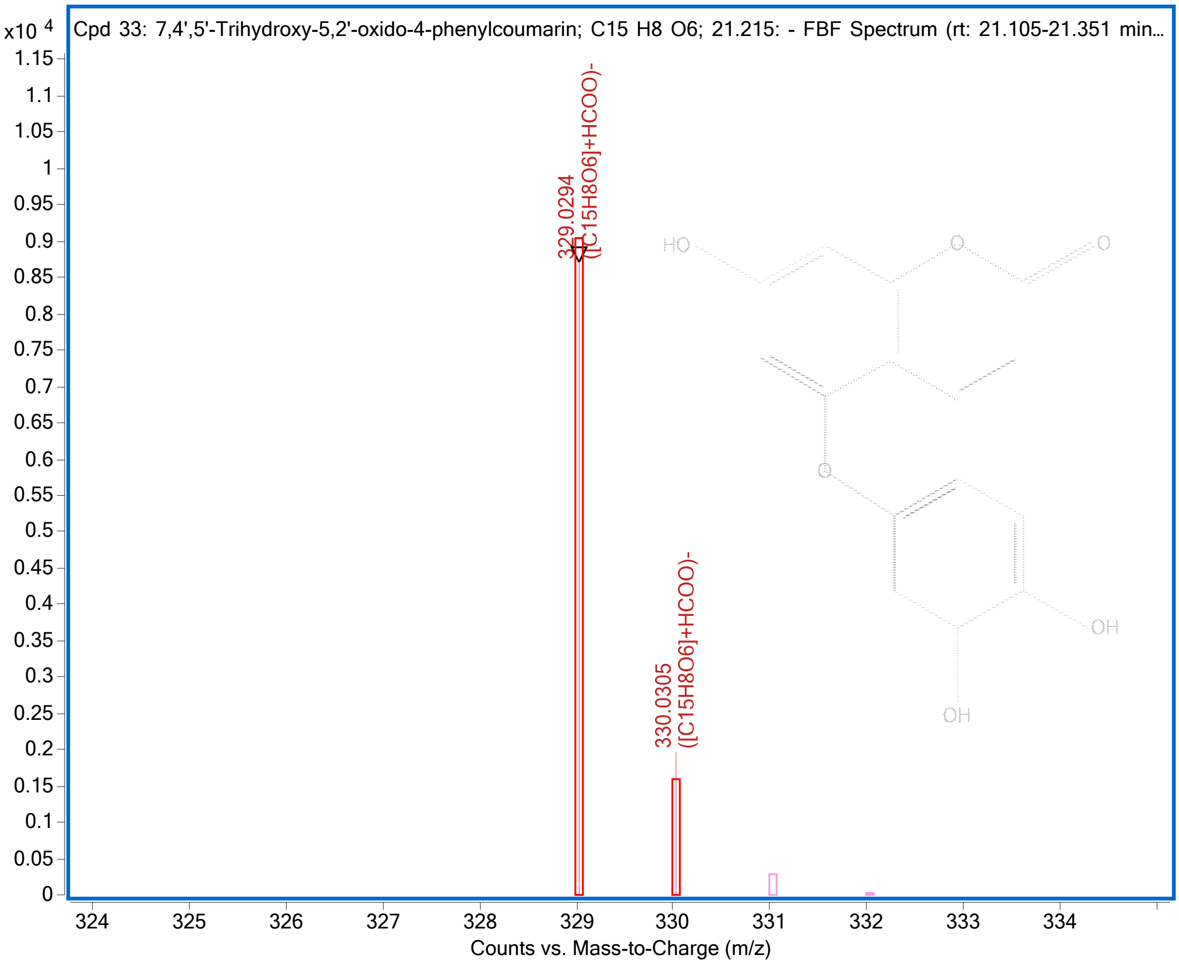

Figure S23. Negative ESI-MS full-scan spectrum of 7,4′,5′-Trihydroxy-5,2′-oxido-4-phenylcoumarin showing the formate adduct ion [M + HCOO]$^{-}$ (m/z 329.0224) formed in the presence of formic acid in the mobile phase.

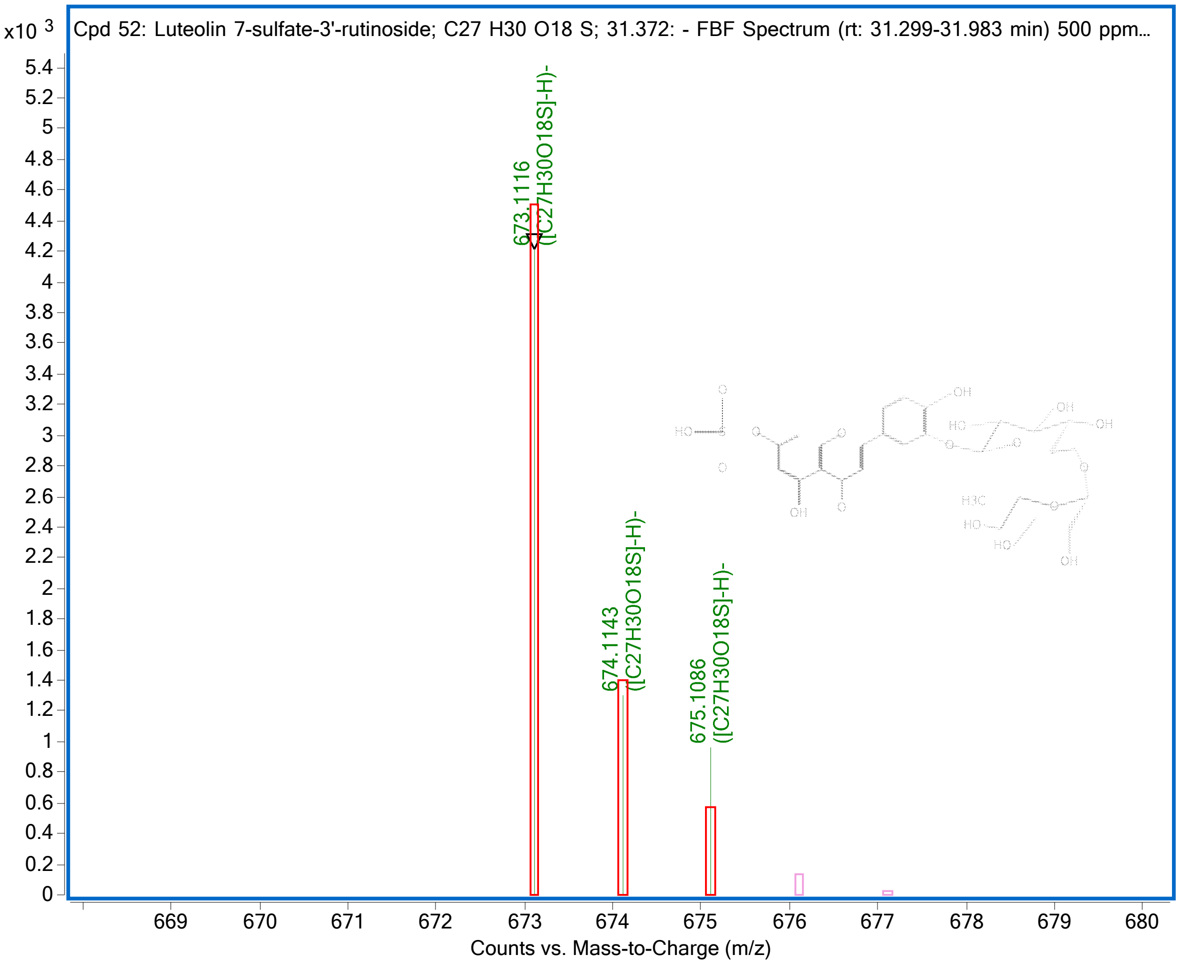

Figure S24. Negative ESI-MS full-scan spectrum of Luteolin-7-sulfate-3′-rutinoside
